# An automated homology-based approach for identifying transposable elements

**DOI:** 10.1186/1471-2105-12-130

**Published:** 2011-05-03

**Authors:** Ryan C Kennedy, Maria F Unger, Scott Christley, Frank H Collins, Gregory R Madey

**Affiliations:** 1 Department of Computer Science and Engineering, University of Notre Dame, Notre Dame, IN, USA; 2 Eck Institute for Global Health, University of Notre Dame, Notre Dame, IN, USA; 3 Department of Biological Sciences, University of Notre Dame, Notre Dame, IN, USA; 4 Department of Surgery, University of Chicago, Chicago, IL, USA

## Abstract

**Background:**

Transposable elements (TEs) are mobile sequences found in nearly all eukaryotic genomes. They have the ability to move and replicate within a genome, often influencing genome evolution and gene expression. The identification of TEs is an important part of every genome project. The number of sequenced genomes is rapidly rising, and the need to identify TEs within them is also growing. The ability to do this automatically and effectively in a manner similar to the methods used for genes is of increasing importance. There exist many difficulties in identifying TEs, including their tendency to degrade over time and that many do not adhere to a conserved structure. In this work, we describe a homology-based approach for the automatic identification of high-quality consensus TEs, aimed for use in the analysis of newly sequenced genomes.

**Results:**

We describe a homology-based approach for the automatic identification of TEs in genomes. Our modular approach is dependent on a thorough and high-quality library of representative TEs. The implementation of the approach, named TESeeker, is BLAST-based, but also makes use of the CAP3 assembly program and the ClustalW2 multiple sequence alignment tool, as well as numerous BioPerl scripts. We apply our approach to newly sequenced genomes and successfully identify consensus TEs that are up to 99% identical to manually annotated TEs.

**Conclusions:**

While TEs are known to be a major force in the evolution of genomes, the automatic identification of TEs in genomes is far from mature. In particular, there is a lack of automated homology-based approaches that produce high-quality TEs. Our approach is able to generate high-quality consensus TE sequences automatically, requiring the user to only provide a few basic parameters. This approach is intentionally modular, allowing researchers to use components separately or iteratively. Our approach is most effective for TEs with intact reading frames. The implementation, TESeeker, is available for download as a virtual appliance, while the library of representative TEs is available as a separate download.

## Background

Transposable elements (TEs) are a type of repetitive sequence that have been found in nearly all eukaryotic genomes. First discovered and analyzed by McClintock in the 1950s [1], TEs have the ability to move and replicate within a genome. Due to their mobile and replicative nature, TEs often occupy large portions of genomes. TEs are estimated to represent 47% of the yellow-fever mosquito genome, *Aedes aegypti *[2], 35% of the frog genome, *Xenopus tropicalis *[3], and 45% of the human genome, *Homo sapiens *[4]. This prevalence of TEs poses a major difficulty in sequence assembly, as repeat regions are prone to misassembly [5,6]. TEs can impact host genomes in a number of ways. They are believed to play a major role in genome evolution [7-9], as they can insert themselves into, mutate, and move genes, thereby influencing gene expression, causing gene variation, and transferring genetic material [10-13].

**Figure 1:**
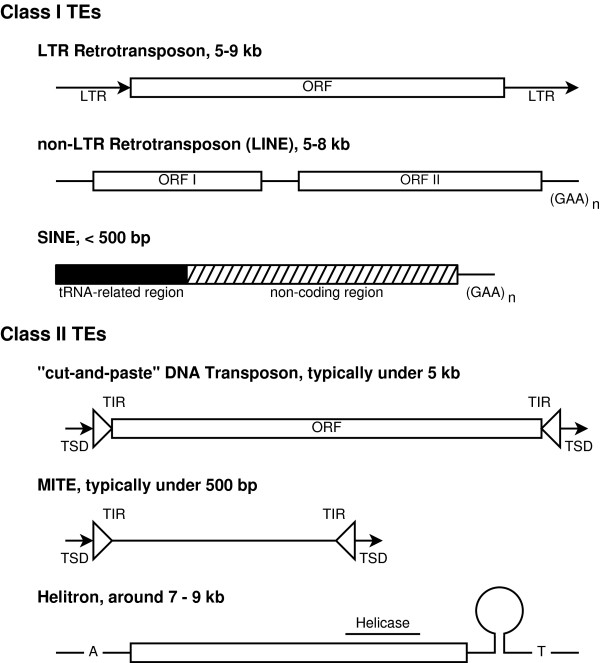
**TE Classification Scheme and Structures**. Adapted from [14], this figure shows the typical division of TE classes, as well as major families within each. This figure is not drawn to scale.

The process by which TEs move about a genome is called transposition. TEs are classified according to their transposition mechanism into Class I and Class II elements. Class I TEs, or retrotransposons, are mediated by an RNA intermediate, typically produced by a TE encoded reverse transcriptase. Retrotransposons transcribe themselves to RNA and are reverse transcribed back into DNA by the reverse transcriptase enzyme, the "copy-and-paste" mechanism. The presence or absence of long terminal repeats (LTRs) further classifies retrotransposons into non-LTR and LTR elements. Class II TEs, or transposons, are DNA-mediated and transpose through the use of a transposase enzyme. Transposons are typically bounded by terminal inverted repeats (TIRs), which flank and serve as the recognition sequence for the transposase. The transposase adheres to a "cut-and-paste" mechanism, as it cuts out the TE from the host DNA and allows it to insert at a new site in the host DNA. Many TEs have preferential insertion sites and the method by which TEs move about genomes often produces artifacts flanking the TEs, called target site duplications (TSDs). Both Class I and Class II TEs are further divided into families, each with distinguishing characteristics. We follow the classification scheme described by Tu [14], summarized in Figure 1.

There are several difficulties with TE identification. TEs do not adhere to a universal structure; rather, some families of TEs follow specific structures. An example would be the TIR-transposase-TIR general structure of a Class II transposon, such as in the *mariner *element. Complicating matters, the structure of TEs can degrade over time. For example, TEs may preferentially insert themselves in similar regions of the genome, or even within one another, leading to many nested and fragmented copies. While autonomous, or active, TEs possess intact reading frames which serve as mechanisms for transposition, the majority of TEs are non-autonomous. Non-autonomous TEs can often still be transposed, using the transcription machinery of other elements in their class. For these reasons, a general approach cannot be used to identify all TEs. Instead, several approaches are used with varying levels of effectiveness.

The automatic identification of TEs is not as robust or mature as analogous methods currently used for genes [15]. Bergman and Quesneville [16] describe many TE discovery methods and classify existing TE discovery techniques into *de novo*, structure-based, comparative genomic, and homology-based discovery methods. Saha et al. and Lerat more recently reviewed approaches to identify TEs [17,18] and classify identification techniques into analogous groups: *ab initio*, signature-based, and library-based techniques. We next describe the approaches according to the Bergman and Quesneville classification.

### *De novo*

*De novo *TE discovery approaches look for similar sequences found at multiple positions within a genome. Once identified, the sequences are typically clustered, filtered, and characterized. While computationally expensive, this approach can identify novel TEs and is most effective in discovering TEs with high prevalence. *De novo *techniques are typically not effective in identifying degraded TEs. Example *de novo *tools include PILER[19] and RECON[20].

### Structure-based

Structure-based approaches, such as LTR_STRUC[21], work well to identify complete TEs that comply to a conserved structure. In this case, LTR_STRUC is effective at finding retrotransposons with LTRs at each end of the element. Structure-based methods are less useful when searching for degraded TEs or for TEs without a conserved structural characteristic, such as LTRs or TIRs.

### Comparative Genomic

A comparative genomic discovery method described by Caspi and Pachter [22] uses multiple sequence alignments of closely related genomes to detect large changes between the genomes. The idea is that differences in the genomes, called insertion regions, could be TEs or caused by TEs. Such differences are analyzed and classified. This approach is useful when related genomes are readily available and can identify new families of TEs. Common ancestral TEs will not be identified by this approach.

### Homology-based

Homology-based approaches utilize known TEs as a means to discover TEs in genomes. This is typically done by manually seeding alignment programs, such as BLAST [23], and then carefully analyzing the results. Biedler and Tu [24] reference a suite of TE-related programs to identify and characterize TEs that are homology-based and Quesneville, et al. offer the BLASTER suite of tools [25] to detect TEs. Although there are few homology-based tools and despite the fact that they struggle in identifying TEs unrelated to known elements, they are normally most accurate in identifying known TEs as well as detecting degraded TEs. Existing homology-based approaches also sometimes utilize hidden Markov models (HMMs) [26], which are effective for closely related genomes, but struggle with distantly related species, as the models tend to capture more irrelevant data when searching for diverse sequences. Additionally, homology-based approaches currently available are the fewest in number [18] and least automated. Moreover, many are not geared to output high-quality consensus sequences.

In this paper, we describe a fast, easy-to-use, and automated homology-based approach to discover high-quality putative TEs, implemented as TESeeker. This approach is aimed to be used in the analysis of TEs in novel genomes.

## Results and Discussion

### TE Library

Our modular homology-based approach relies on a thorough and high-quality library of representative TEs, organized by family. When strong information is available, amino acid coding regions, reverse transcriptases for Class I TEs and transposases for Class II TEs, are the preferred components of the library. Nucleotide sequences can also be used, but such sequences do not allow for as much nucleotide variance during the search. Sequences for our library were chosen manually from TEfam, [27], NCBI [28], Repbase [29], and the literature. Sequences with intact amino acid coding regions were preferentially chosen and a wide variety of related sequences was assembled for each family. Currently, the library consists of 475 representative coding regions from a variety of (largely arthropod) organisms and covers the major TE families. For Class I elements, the library consists of 227 LTR amino acid sequences representing the *cer1, copia, csrn1, Cyclops, gypsy, mag, mdg1, mdg3, osvaldo, Pao/Bel, and Ty3 *families as well as 49 non-LTR amino acid sequences representing the *CR1, I, Jockey, L1, L2, LOA, Loner, Outcast, R1, R4*, and *RTE *families. The library consists of 199 amino acid sequences for Class II elements, namely the *gambol, hAT*, *mariner, p, piggyBac, pogo, and Tc1 *families. Further details on the provided library are available within the FASTA files and online [30]. Because the library consists of sequences in the FASTA format, researchers can easily modify the library or create their own library for use in the approach.

### Approach

**Figure 2:**
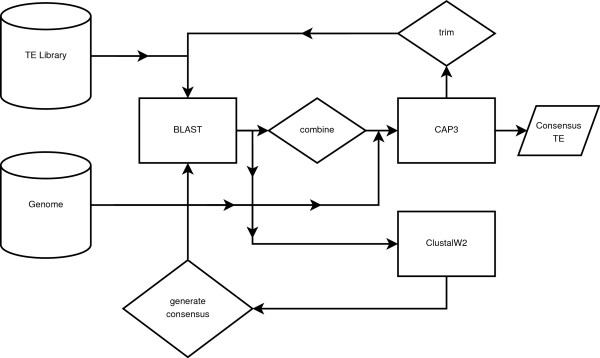
**Approach Schematic**. The approach is composed of multiple, iterative steps. The general flow is as follows. A TE family is used in a BLAST search against the genome. Hits are then combined, extracted from the genome, and assembled with CAP3. Next, the sequences are trimmed and again used in a BLAST search against the genome. The results are then used to produce a multiple sequence alignment in ClustalW2. We generate a consensus from the alignment, and then perform a final BLAST search against the genome. We again combine, extract, and assemble with CAP3. Lastly, the consensus TE is generated from CAP3.

Our approach varies slightly depending on whether the representative TEs are amino acid or nucleotide sequences, the main difference being that amino acid searches require only a translated nucleotide genome search, tblastn, while nucleotide sequences require translation of both themselves and the host genome, tblastx. We next describe the approach that starts with an amino acid library of TEs, shown graphically in Figure 2.

The approach begins with BLAST searches against the genome using representative TEs for the chosen family. Resulting BLAST hits are combined if they overlap or are very close together, and are then extracted from the genome. We next assemble with CAP3 in an attempt to gain a viable representation of the coding sequence. We use the CAP3 results to do another BLAST search against the genome and process the hits in the same manner. However, when extracting the sequences from the genome, we add flanking regions. The length of the flanking region is dependent on the type of TE and is utilized to enable us to capture the entire TE. These results are then aligned and a consensus is generated. We use the consensus to perform a final BLAST search, again combining, extracting, and assembling the sequences. CAP3 then produces the high-quality, full-length consensus TE. We next describe the approach in more detail.

#### Identify Coding Region

**Figure 3:**
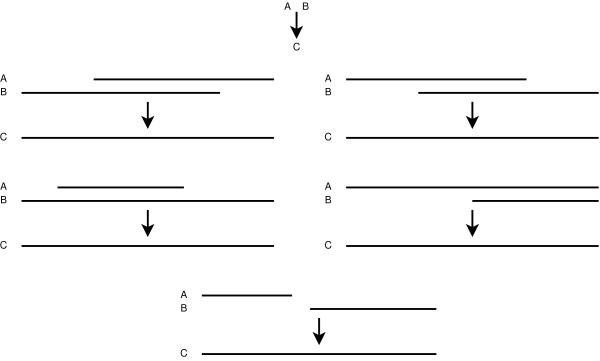
**Methods of Combination**. This figure shows the five general combination scenarios used in our scripts. In each case, hit sequences A and B are consolidated into a single sequence C, which represents a section of nucleotides from the genome. We have shown combinations of overlaps, nested sequences, and sequences separated by a short, prespecified distance.

The coding region is generally most conserved across TEs within a genome, as it must be complete to produce a functional protein. We begin with local sequence alignments using BLAST. Nucleotide-based blastn searches are not as effective in identifying TEs and are not used; the nucleotide sequence for a given TE may vary considerably, while the translated amino acid sequence is more likely to be conserved. Instead, tblastn searches are used to identify the coding region. BLAST produces a set of hits for each TE query against the genome and we consider hits with an e-value less than 1e-20 for our approach. This cutoff was determined from our empirical data to limit the hits to the most probable TEs while also eliminating most false positives and can also be manually adjusted. Due to slight sequence variations, BLAST results are often rich in short, nearly-adjacent hits. We process BLAST results such that hits are combined if they are within a specified distance of one another, 50 bp by default, and originate from the same query sequence. Hits with overlapping coordinates are combined as well. These combinations increase the quality of our hits and the potential to capture more complete sequences. In the case where there is a gap between sequences, we also include the intervening sequence data in our hit. Figure 3 shows combination scenarios. Once all possible combinations are performed, hits are extracted from the genome.

At this point, we have a set of possible coding sequences, both complete and partial, many of which are copies or partial copies of one another. To consolidate and improve our results, we assemble the sequences with the CAP3 assembly program [31]. CAP3 produces a set of consensus sequences generated from multiple sequences, called contigs, as well as sequences that did not assemble with other sequences, called singlets. CAP3 also generates accompanying quality scores for the contig sequences. We use the quality scores to trim the sequences such that the highest quality sequence remains. To do this, we iterate through a contig, keeping track of the cumulative sum of quality scores for a given number of consecutive nucleotides, called the sliding window, which is 20 bp by default. When the average value of a nucleotide in this sliding window exceeds a threshold, 18 by default, we consider the corresponding sequence to be high-quality. If the average value drops below the threshold, the sequence is ignored. Once we have read the entire sequence, there will likely be gaps in the sequence where there is little commonality. In these cases, we only keep the low-quality regions if they are of short length and have adjacent high-quality sequences. These results are then reassembled in CAP3, trimmed, and considered the best potential complete coding region. In the case that CAP3 produces only singlet sequences, we perform the aforementioned analysis with them. We then extend the sequence to encompass the entire TE. Pseudocode for the steps described in this section of our approach is shown in Algorithm 1.

#### Encompass Complete TE

Once the putative coding region has been identified, we create a consensus for the complete TE. We perform a blastn search with each contig from the previous (CAP3) step attempting to find the instances of the TE within the genome. We again process these hits as before and extract them from the genome, but this time we also extract flanking regions on either side of the viable hits in an attempt to capture the entire TE. This extracted set of instances can then be used to generate a consensus sequence.

#### Generate Consensus

The extracted near full-length sequences from the previous step are inherently very similar on a nucleotide-by-nucleotide basis. To generate a consensus from this set of sequences, we perform a multiple sequence alignment with ClustalW2 [32]. A consensus sequence is generated as follows from the multiple sequence alignment. We record counts for each nucleotide at each position in the alignment file. If a gap is encountered, counts for each nucleotide are incremented. If the percentage for any nucleotide at a given position exceeds a given threshold, 49% by default, that nucleotide is used for that position in the consensus. We now have a consensus sequence for the TE that is the most likely sequence to occur in the genome and we need to verify that it is complete.

#### Identify Complete TE

To validate and improve the consensus sequence, we look for similar copies of it in the genome with a blastn search. We again process the BLAST hits as previously described and extract them from the genome, generally adding short flanking sequences. The resulting extracted sequences are again iteratively examined with CAP3 and trimmed. CAP3 produces a sequence which represents the best estimate for a consensus putative TE in the novel genome. Further inspection on the putative TE is advisable, both in terms of validity and classification. Once validated, this TE can be utilized to calculate the density of its particular family within the genome and to find individual instances.

### Implementation

Our approach is implemented as TESeeker and was purposely designed to be modular, while relying upon common bioinformatics tools, namely BLAST, CAP3, and ClustalW2, as well as BioPerl [33]. TESeeker is released as a VirtualBox [34] virtual appliance. The local web browser interface to TESeeker offers the main gateway to the core TESeeker functionality; however, TESeeker can also be run through the command line. A researcher needs to only provide basic parameters, such as TE family, host genome, closeness to combine, flank length, CAP3 window size, and the CAP3 quality score threshold for consensus generation. Suggested starting parameters include combining BLAST hits within 50 bp, a CAP3 window size of 20 bp, a combine distance of 50 bp, and a CAP3 quality score threshold of 18. These parameters were determined through extensive testing on arthropod genomes. Further details on suggested parameters, as well as means to perform a sample run are provided within the virtual appliance. While not parallelized, researchers can easily run multiple instances of TESeeker while varying parameters and TE families, offering scalability.

### Testing

This approach was developed over the course of several TE detection projects on several arthropod genomes [35,36], but was not originally automated. DNASTAR SeqMan II [37] was used in place of CAP3 and ClustalW2. DNASTAR SeqMan II produced viable results, but it required extensive interaction from a researcher. Sequences had to be manually examined and trimmed in the program, a process which took considerable time and required a trained researcher. This manual approach produced results that we consider a high-quality annotation of TEs. We used these results to partially validate TESeeker against the *Pediculus humanus humanus *genome, described later. We also evaluated our approach against published results from the *Anopheles gambiae *PEST genome, as well as a number of other genomes. We utilized our library of representative coding regions for validation. Except when we used TESeeker to reconstruct an element from its amino acid coding region, we removed all sequences in the library that originated from the genome in question.

#### *Pediculus humanus humanus *Genome

The body louse, *Pediculus humanus humanus*, is the primary vector of typhus and several other diseases [38]. It is the smallest presently sequenced insect genome at roughly 110 Mb. TESeeker was able to identify all Class I and Class II TEs, with the exception of MITEs, reported in Kirkness et al. [35]. Unlike many other arthropod genomes, only 1% of the *P. humanus humanus *genome is made up of TEs.

As reported in Kirkness et al. [35], there were 4 families of TEs identified in *P. humanus humanus*, 3 Class I elements and 1 Class II element [35]. The *ty3/gypsy *LTR retrotransposon is well-represented in the genome, but there are only 2 full-length copies. Non-LTR elements belonging to the *SART *and *R4 *families are also present, each also with a small number of full-length elements. The Class II *mariner *element is much smaller, but there are 24 full-length copies in the genome. Detailed results for these elements are described in Table 1. Our approach was successful in detecting each of these TEs.

**Table 1:** *Pediculus humanus humanus* Results

Class I	Family	Element	Length (bp)	Full-length Copies	Copies	Density
non-LTR						
	SART	*Hope-like*	4655	1	522	0.18%
	R4	*Dong-like*	5266	4	1739	0.45%
LTR						
	ty3/gypsy	*Mdg1*	5395	2	976	0.28%

**Class II**	**Family**	**Element**	**Length (bp)**	**Full-length Copies**	**Copies**	**Density**

	mariner/Tc1	*mariner*	1276	24	216	0.09%

TOTAL						1.00%

This table shows information for the TEs identified in *P. humanus humanus*. We identified 4 families of TEs, with full-length copies of each present in the genome. Results were previously reported in Kirkness et al. [35].

TESeeker correctly identified all previously reported TEs in *P. humanus humanus*. Running TESeeker with suggested parameters for a *mariner *Class II element in *P. humanus humanus *produced a consensus TE that was 99% identical to the manually annotated element. Additionally, the ends were well-trimmed. The alignment for this comparison can be found in Additional file 1. Modifying the parameters to account for differences in TE characteristics, TESeeker identified the Class I TEs as well. We detected the *Dong-like *TE, flanked by its TSDs almost perfectly. We also correctly detected the *ty3/gypsy *TE, with 60 bp of extra sequence on either end, as well as the *Hope-like *TE. Our approach's ability to discover TEs of varying families, across classes, in a genome with so few TEs demonstrates its utility. Partial or degraded copies within *P. humanus humanus *were found through BLAST searches using the full-length consensus sequences as queries.

#### *Anopheles gambiae* PEST Genome

*Anopheles gambiae *serves as the main vector of malaria [39]. The PEST strain is roughly 273 Mb and has been extensively studied. Class II *P *elements within the genome have been especially closely examined. Sarkar et al. originally identified 6 distinct *P *elements [40]. More recently, Oliveira de Carvalho et al. identified 4 additional *P *elements [41], while Quesneville et al. described 9 clades that are at least 30% divergent at the nucleotide level [42]. In all, previous research has described 12 clades of *P *elements in *A. gambiae *that are more than 30% divergent at the nucleotide level.

TESeeker detected 11 out of the 12 *P *elements within *A. gambiae*, as well as an additional 2 partial hits that showed strong similarity to *P *element transposase, but that were more than 30% divergent at the nucleotide level. The lone element that TESeeker missed, AgaP14, is most divergent from the other elements, which may explain its absence and which also suggests our library does not fully represent the *P *element family. Additionally, TESeeker produced consensus sequences with TIRs on every element where they had been previously reported.

Searches for additional Class II TE families were also successful. In particular, we identified 10 of the 13 *piggyBac *elements, with TIRs when present, described in Sarkar et al. [43]. Again, the elements TESeeker missed were most divergent from the other sequences. TESeeker did especially well with *mariner *elements. TESeeker identified each of the 5 elements at TEfam, each with complete TIRs and 4 with the expected TSDs.

Further testing was performed on Class I TEs available on TEfam. To validate the ability of our approach to reconstruct a full-length TE from a given coding region, we populated our library with coding regions from Class I TEs. For 18 of the 19 full-length non-LTR elements on TEfam with amino acid coding regions available and also with full-length copies present in the genome, TESeeker successfully reproduced the full-length element. Parameters for the CAP3 window quality often needed to be relaxed, as some sequences present in TEfam are not abundant in the genome. Results from TESeeker were typically slightly longer on both ends, due to relaxed parameters. The single element TESeeker did not produce in its entirety, *Loner*, was still over 90% reconstructed with more than 80% identity to the TEfam element. Additional file 2 provides ClustalX alignments for one member of each non-LTR family from TEfam against TESeeker-produced full-length elements. TESeeker was also able to reconstruct over 99% of the composite *Pao/Bel *LTR element from TEfam with 98% identity. In addition to the TEs found in TEfam, TESeeker produced many additional singlet and contig sequences because of the relaxed parameters. While extensive validation was not performed on these sequences, many appear to have intact coding regions and are potentially TEs present in the genome but not in the TEfam database.

#### Other Organisms

TESeeker was also validated on select elements in a variety of organisms. Of particular note, we detected a previously unreported putative *mariner *element in the well-studied *Drosophila melanogaster *genome. The 1061 bp element has TIRs 26 bp in length, with 3 mismatches, but with no apparent TSDs. A single full-length copy, as well as a small number of partial hits, exist within the genome. Its transposase has a high homology to related insects, such as *Chymomyza amoena *and *Cladodiopsis seyrigi*. Searches for this element in existing TE annotations for *D. melanogaster *produced no hits. Please refer to Additional file 3 for an annotated version of this putative element.

Additionally, TESeeker was used to search for *mariner *elements in the human (*Homo sapiens*), frog (*Xenopus tropicalis*), and chicken (*Gallus gallus*) genomes. *Mariner *elements are known to exist in the human, frog, and chicken genomes, which were found using TESeeker.

### Advantages

Our approach offers many advantages to researchers. First, TESeeker allows for the fast and accurate detection of TEs. As demonstrated in several genomes, across multiple TE families, TESeeker effectively identifies TEs. In addition to TE identification, our approach offers opportunities to reexamine and validate previous research. Second, TESeeker is very easy to use; we provide TESeeker as a virtual appliance, completely configured. Researchers need only provide a few parameters to begin searching. Parameters are easily modified and multiple iterations of the approach can be run simultaneously. Third, TESeeker is general. While we primarily evaluated our approach on TEs in arthropod genomes, the parameters can be adjusted to allow for the effective detection of a variety of TE families in any genome, including genomes that contain only degraded TEs. Less stringent parameters will be more effective in detecting such degraded TEs, but will also increase the number of false positives. As mentioned previously, we have utilized various stages of this approach to identify non-LTR and LTR TEs in a number of genome projects. Last, our approach eases the burden on expert annotators, decreasing genome annotation time.

### Limitations

While robust, this approach has several limitations. First, results are highly dependent on the quality of the sequences in the library and whether the novel genome contains TEs with homology to those in the library. The library must contain a thorough representation of TEs for a given family, preferably amino acid coding regions. The provided library has performed well, but extensive testing has not been performed on LTR elements. Additionally, this approach is not designed to detect TEs without a coding region, such as SINEs or MITEs. Second, the approach is most effective for TEs that exist in multiple copies throughout the genome. While TESeeker has been shown to find TEs that have only a single full-length instance, the quality of its output and the extra effort required by the researcher to alter the parameters can be time-consuming. Last, results from TESeeker must be closely examined. An ongoing issue with TEs concerns their classification. If a search is seeded with *mariner *sequences, it may produce consensus TEs that are not true *mariners*, but are rather *mariner*-like TEs. For this manuscript, TEs were classified through manual examination of their amino acid coding regions.

## Conclusions

The number of sequenced genomes is rapidly increasing and the necessity to annotate them effectively and quickly is more important than ever. TEs are an important evolutionary force present in the majority of these genomes. While there are mature, effective, and automated gene identification systems, the tools available for TEs are not as robust. Particularly, current homology-based approaches are typically very interactive, requiring numerous user decisions and the manual start of separate tools.

The approach described herein successfully identifies TEs in novel genomes in an automated and user-friendly package, offering researchers the ability to quickly produce high-quality consensus TEs. TESeeker was developed and refined over the course of several TE identification projects and works best to detect TEs with homology to known TEs. We are able to generate high-quality putative TEs as well as characterize the prevalence of TEs in many genomes. We provide TESeeker as a web-based tool within a virtual appliance, while also providing our representative TE library. While its local web interface automates the underlying logic, each step can be manually started through the command line, offering additional flexibility. Our approach's ability to automatically analyze a genome alleviates the exhaustive, error-prone, and time-consuming task of manually inspecting and manipulating results. The performance of TESeeker varies, but is largely dependent on the length of the TE family and its abundance in the genome.

## Future Work

Due to the nature of TEs, there will likely never be an all-encompassing approach for their detection. Instead, existing approaches will be improved and used in conjunction with other approaches. With TESeeker, several improvements could be implemented. First, incorporating the ability to detect structural characteristics in TEs, such as LTRs and TIRs, would allow us to more correctly trim our results. Second, the ability for TESeeker to automatically determine the length of the required flanking sequence based on family characteristics would require less expertise on the part of the researcher. This would be especially useful when coupled with classification techniques that could be applied to the results from TESeeker. Third, TESeeker could utilize additional tools to detect TEs without coding regions, such as MITEs or SINEs. Fourth, as mentioned previously, the results of TESeeker will only be as good as the sequences in the library. Namely, TESeeker will not find TEs without homology to those within the library. Therefore, we will provide updates to the library online. Last, we could implement a cross-validation routine which would learn the optimal parameter settings for a given family, further easing the burden on the researcher.

## Availability and Requirements

TESeeker is available as a VirtualBox virtual appliance running 32-bit Ubuntu 10.04 LTS with all scripts and tools configured. Documentation and the representative library are available as separate downloads. Please see Additional file 4 for the complete user manual, which also features an example search walkthrough.

**Project name**: TESeeker

**Project home page**: http://repository.library.nd.edu/view/27/teseeker

**Operating systems**: Windows, OS X, Linux, Solaris

**Programming languages**: Perl, bash

**Other requirements**: VirtualBox

**License**: GNU General Public License (GPL) v3

**Any restrictions to use by non-academics**: none

## Authors' contributions

All authors contributed to the approach's design. RCK and SC designed and implemented the code. RCK tested the approach on various genomes and designed the virtual appliance. MFU and RCK assembled sequences for the representative library. All authors discussed and analyzed the biological data. RCK drafted the manuscript and all authors read, edited, and approved the final manuscript.

## Algorithm 1

P = IDENTIFYPUTATIVESEQUENCES (*Q, S, evalue, distance*)

Let *Q *be the set of representative TEs

Let *S *be the genome

Let *P *be the set of putative hits

Let *evalue *be the maximum e-value of a potential hit

Let *distance *be the maximum distance between potential hits

// Search genome and sort hits according to location

**for all ***q *∈ *Q ***do**

   *H_q _*← BLAST(*q*, *S*)

   *H_q _*← sort(*H_q _*, *position*)

end for

// Combine overlapping hits

**for all ***q *∈ *Q ***do**

   **for all ***h *∈ *H_q _***do**

      **if ***h *≤ *evalue ***then**

         **for all ***i *∈ *H_q _***do**

            **if ***i *≤ *evalue ***then**

               **if **abs(*h.location - i.location*) ≤ *distance ***then**

                  *h *← (*h *+ *i*)

               **end if**

            **end if**

         **end for**

      **end if**

   **end for**

end for

// Extract putative TEs from genome

**for all ***q *∈ *Q ***do**

   **for all ***h *∈ *H_q _***do**

      *P_q _*← extract(*h*, *S*)

   **end for**

end for

// Assemble consensus TEs

**for all ***p *∈ *P_q _***do**

   p ← trim(CAP3(*p*))

end for

**return ***P*

## Supplementary Material

Additional file 1 **ClustalX ****alignment of the manually annotated *mariner* and the ****TESeeker****-produced *mariner*, both from *****P. humanus humanus***. Click here for file

Additional file 2 **ClustalX**** alignments of selected *A. gambiae* PEST non-LTR elements from TEfam and the ****TESeeker****-produced full-length elements**. Click here for file

Additional file 3 **Annotated putative *D. melanogaster mariner* element**. Click here for file

Additional file 4 **TESeeker**** User Manual.** Click here for file
