# Supplementary material for: An automated homology-based approach for identifying transposable elements
Source: BMC Bioinformatics. 2011 May 3;12:130. doi: 10.1186/1471-2105-12-130 (PMC3107183; doi:10.1186/1471-2105-12-130)
Supplement: Additional file 1 — ClustalX alignment of the manually annotated mariner and the TESeeker-produced mariner, both from P. humanus humanus. [file 1471-2105-12-130-S1.PDF]

Additional File 1

`ClustalX` alignment for the *Pediculus humanus humanus mariner* element. Here, we show the manually annotated *mariner* element aligned with the `TESeeker`-produced element.

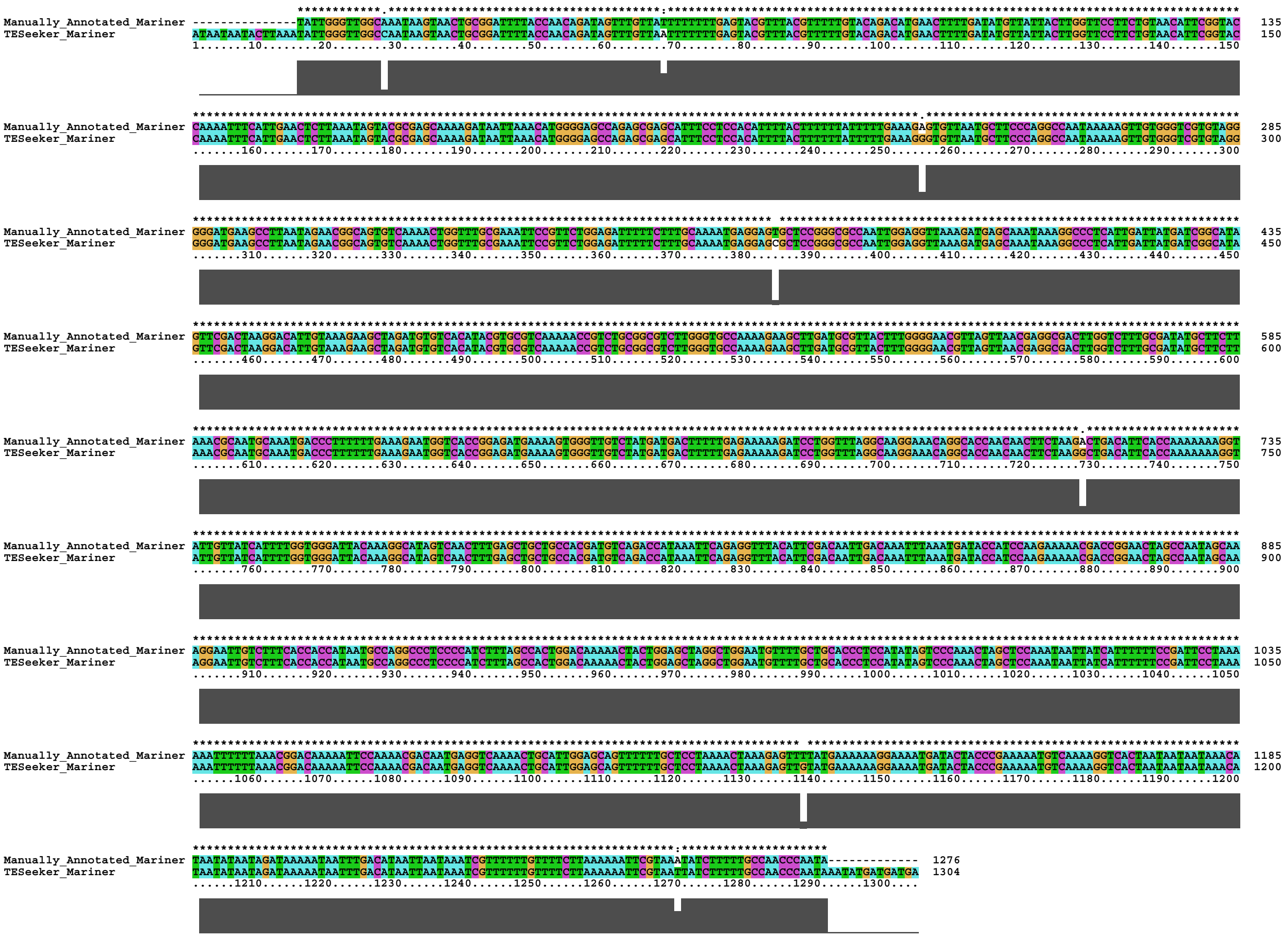

Manually_Annotated_Mariner
TESeeker_Mariner
135
150
285
300
435
450
585
600
735
750
885
900
1035
1050
1185
1200
1276
1304
