# Supplementary material for: An automated homology-based approach for identifying transposable elements
Source: BMC Bioinformatics. 2011 May 3;12:130. doi: 10.1186/1471-2105-12-130 (PMC3107183; doi:10.1186/1471-2105-12-130)
Supplement: Additional file 2 — ClustalX alignments of selected A. gambiae PEST non-LTR elements from TEfam and the TESeeker-produced full-length elements. [file 1471-2105-12-130-S2.PDF]

Additional File 2

ClustalX alignments for *Anopheles gambiae* PEST non-LTR elements. The following 6 alignments show non-LTR elements from TEfam aligned with TESeeker-produced full-length elements. In each case, we were able to fully reconstruct the element.

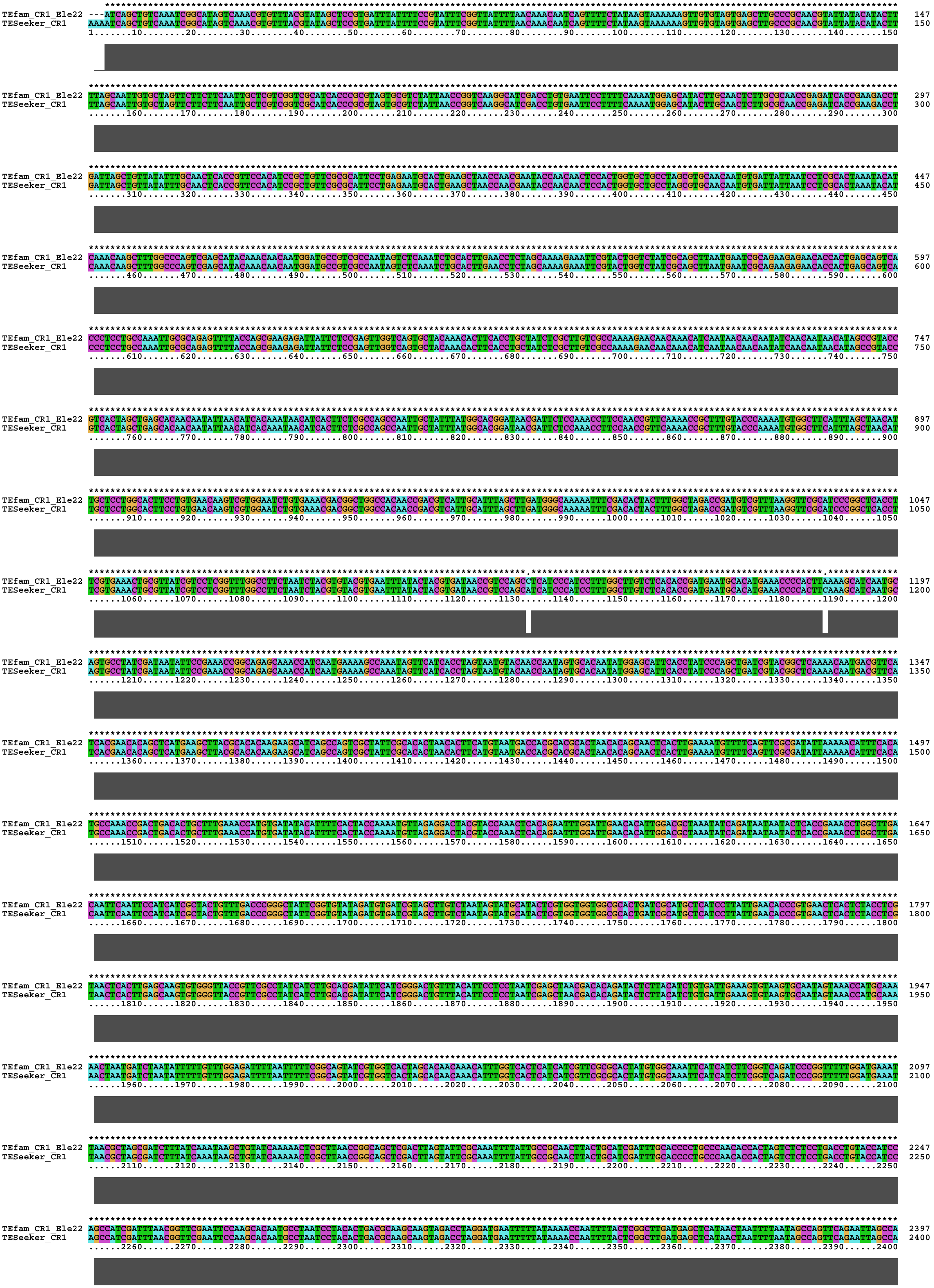
TEfam_CR1_Ele22
TESeeker_CR1

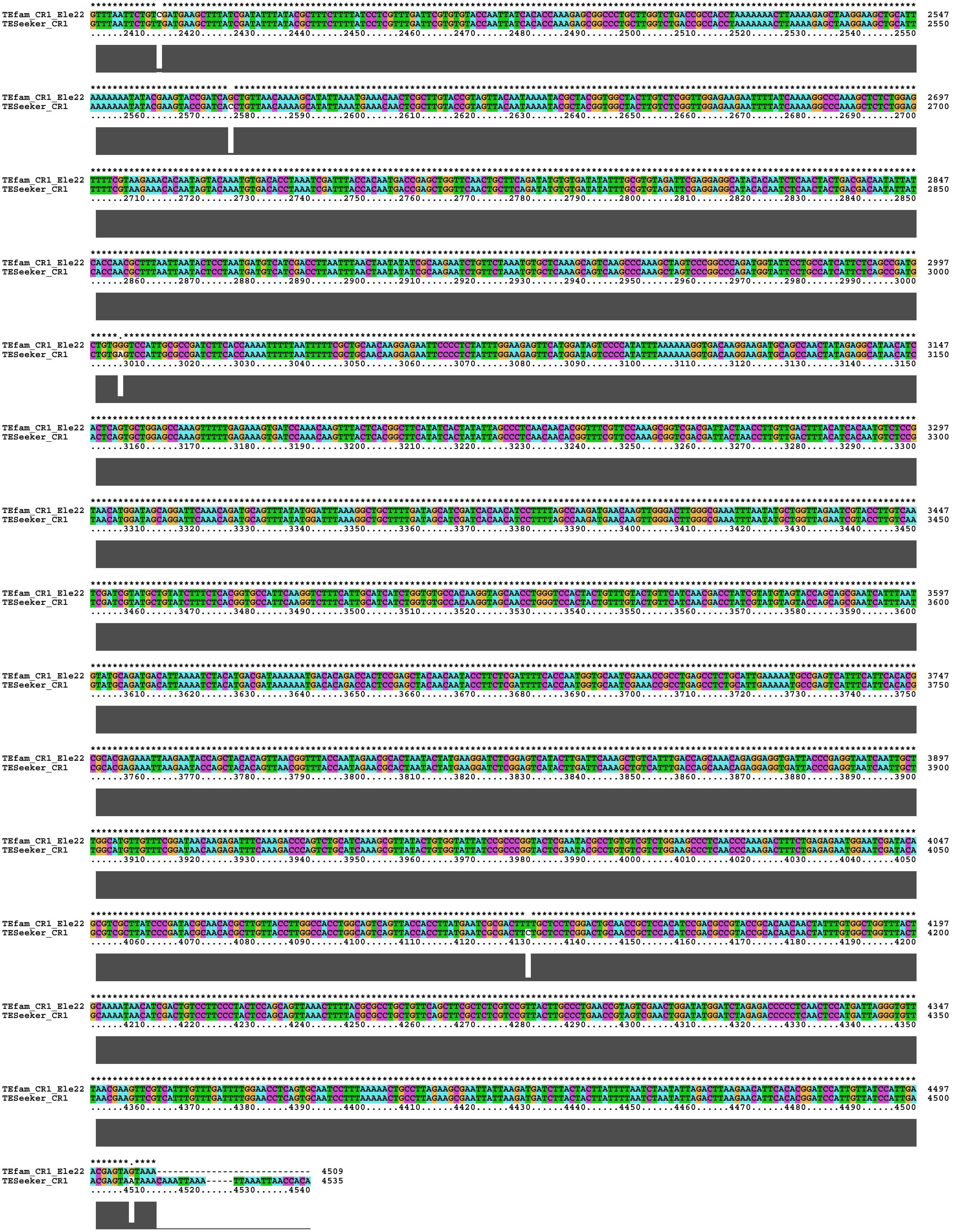
TEfam_CR1_Ele22
TESeeker_CR1

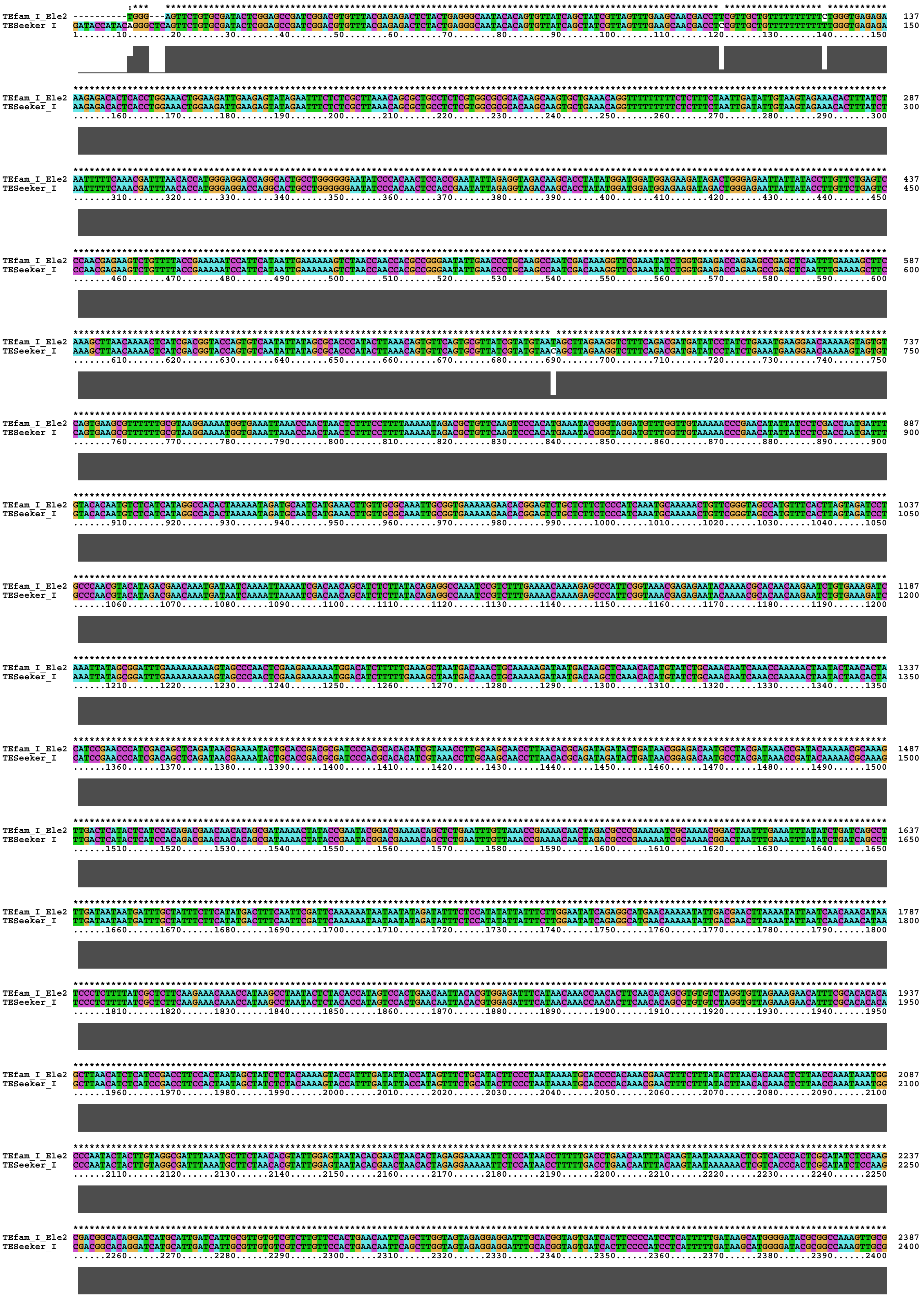
TEfam_I_Ele2
TESeeker_I
137 150
287 300
437 450
587 600
737 750
887 900
1037 1050
1187 1200
1337 1350
1487 1500
1637 1650
1787 1800
1937 1950
2087 2100
2237 2250
2387 2400

```
              ******************************************************************************************************************************************************
TEfam_I_Ele2  ACCACGTTGGAAATATAATGAGGCTGATTGGTCTAGTTATCGTTTCCTTATCGACAGGGCTATTGATGGGTCAGCACTGCTTTCGGTTGATAGTTTTTGCAGTATTATTCTTATGGCAGCAGAACTAAGCATCCCACGAACGTCAGGCAA  2537
TESeeker_I    ACCACGTTGGAAATATAATGAGGCTGATTGGTCTAGTTATCGTTTCCTTATCGACAGGGCTATTGATGGGTCAGCACTGCTTTCGGTTGATAGTTTTTGCAGTATTATTCTTATGGCAGCAGAACTAAGCATCCCACGAACGTCAGGCAA  2550
              ......2410......2420......2430......2440......2450......2460......2470......2480......2490......2500......2510......2520......2530......2540......2550

              ******************************************************************************************************************************************************
TEfam_I_Ele2  AATTCCCAAAAAGTCGGTACCATGGTGGAATGACTCGGTTGAAAAAGCAATAACAGCACGAAGAAAAGCTCTACGGAAATTGAGGAAAGCCAGCACTATCAGTAACAACCCGAATACTTCATACCTAGCTTTGAAGTACCAAGAAGCAAA  2687
TESeeker_I    AATTCCCAAAAAGTCGGTACCATGGTGGAATGACTCGGTTGAAAAAGCAATAACAGCACGAAGAAAAGCTCTACGGAAATTGAGGAAAGCCAGCACTATCAGTAACAACCCGAATACTTCATACCTAGCTTTGAAGTACCAAGAAGCAAA  2700
              ......2560......2570......2580......2590......2600......2610......2620......2630......2640......2650......2660......2670......2680......2690......2700

              ******************************************************************************************************************************************************
TEfam_I_Ele2  CCAGATAGCCAAAAATGAAATAGAAAAAGCAAAAAAAGTAAGTTGGGAAAAATTCGTCACAAGCATTGACCCATCTCTTTCATGCAAAGAAGTTTGGAGTATGGTGAAAAATTTGACAGGAAATAGTCAGTTCTCATTTCCAACACTGTT  2837
TESeeker_I    CCAGATAGCCAAAAATGAAATAGAAAAAGCAAAAAAAGTAAGTTGGGAAAAATTCGTCACAAGCATTGACCCATCTCTTTCATGCAAAGAAGTTTGGAGTATGGTGAAAAATTTGACAGGAAATAGTCAGTTCTCATTTCCAACACTGTT  2850
              ......2710......2720......2730......2740......2750......2760......2770......2780......2790......2800......2810......2820......2830......2840......2850

              ******************************************************************************************************************************************************
TEfam_I_Ele2  GAACAACAACGAACCAATCACTTCTCCATCCTGCATTGCAGAACATTTTGCACAACATTTCTATGAGGCATCTGCAACTTCTAAATACTCAGCTACCTTCATTCAACGCCACAAATCTAAAACTATATCATCCATATCATTTGTTGATAC  2987
TESeeker_I    GAACAACAACGAACCAATCACTTCTCCATCCTGCATTGCAGAACATTTTGCACAACATTTCTATGAGGCATCTGCAACTTCTAAATACTCAGCTACCTTCATTCAACGCCACAAATCTAAAACTATATCATCCATATCATTTGTTGATAC  3000
              ......2860......2870......2880......2890......2900......2910......2920......2930......2940......2950......2960......2970......2980......2990......3000

              ******************************************************************************************************************************************************
TEfam_I_Ele2  AAATAATCAATCGTATAACAAACCATTTTCCACAGAAGAACTAATCTGGGCTTTGAGAAAATGTAGAGGAAACTCTGCGGGTATAGACGATATCGGCTATCCACTCCTCAAGCAGCTTCCTGAGCGTGCATTACACTGCCTTCTAGAAAT  3137
TESeeker_I    AAATAATCAATCGTATAACAAACCATTTTCCACAGAAGAACTAATCTGGGCTTTGAGAAAATGTAGAGGAAACTCTGCGGGTATAGACGATATCGGCTATCCACTCCTCAAGCAGCTTCCTGAGCGTGCATTACACTGCCTTCTAGAAAT  3150
              ......3010......3020......3030......3040......3050......3060......3070......3080......3090......3100......3110......3120......3130......3140......3150

              ******************************************************************************************************************************************************
TEfam_I_Ele2  ATATAACAACATATGGAGGACAGGTGAAATTCCAAATAATTGGAAAACTAGTCTGATCGTCCCAATTCCAAAGGCAGAAAAAGACAGCTTAAGTGTAGACAGTTATCGACCAATCTCTTTGCTGTGTTGTATGAGTAAAGTCCTGGAACG  3287
TESeeker_I    ATATAACAACATATGGAGGACAGGTGAAATTCCAAATAATTGGAAAACTAGTCTGATCGTCCCAATTCCAAAGGCAGAAAAAGACAGCTTAAGTGTAGACAGTTATCGACCAATCTCTTTGCTGTGTTGTATGAGTAAAGTCCTGGAACG  3300
              ......3160......3170......3180......3190......3200......3210......3220......3230......3240......3250......3260......3270......3280......3290......3300

              ******************************************************************************************************************************************************
TEfam_I_Ele2  TTAGTCAATCGACGACTAATTCAGGAGCTAGAGAGCAGAAATCTGTTAAGTGACAAACAGCACGCTTACAGAATCGGCCGTGGTACAGAGACTTATTTTGCTTGTTTAGAAAACGTTCTCACTGAGGCTAAAAATAAAAATTATCACAC  3437
TESeeker_I    TTAGTCAATCGACGACTAATTCAGGAGCTAGAGAGCAGAAATCTGTTAAGTGACAAACAGCACGCTTACAGAATCGGCCGTGGTACAGAGACTTATTTTGCTTGTTTAGAAAACGTTCTCACTGAGGCTAAAAATAAAAATTATCACAC  3450
              ......3310......3320......3330......3340......3350......3360......3370......3380......3390......3400......3410......3420......3430......3440......3450

              ******************************************************************************************************************************************************
TEfam_I_Ele2  TGATTGTGCAGTTGTTGACCTATCAAAGGCCTTCGATCGTACCTGGCGTCCGGCGATCCTTGAGCAATTGAAGAAATGGGGATTTGGAGGAAGTTTACCGCGCTTTATAAAAAATTTCCTAACAGACCGGACCTGTAAAGTACTGATTGG  3587
TESeeker_I    TGATTGTGCAGTTGTTGACCTATCAAAGGCCTTCGATCGTACCTGGCGTCCGGCGATCCTTGAGCAATTGAAGAAATGGGGATTTGGAGGAAGTTTACCGCGCTTTATAAAAAATTTCCTAACAGACCGGACCTGTAAAGTACTGATTGG  3600
              ......3460......3470......3480......3490......3500......3510......3520......3530......3540......3550......3560......3570......3580......3590......3600

              ******************************************************************************************************************************************************
TEfam_I_Ele2  ATCATATACCTCTAACATCAAGGTTTTGGAGAACGGTGTCCCTCAAGGTGCTATATTATCACCTACGCTTTTTCTAATAAGCATGGAGTCTTTATTACAAACGATACCTACTAATATAGACACATTCATTTATGCTGATGACATTATACT  3737
TESeeker_I    ATCATATACCTCTAACATCAAGGTTTTGGAGAACGGTGTCCCTCAAGGTGCTATATTATCACCTACGCTTTTTCTAATAAGCATGGAGTCTTTATTACAAACGATACCTACTAATATAGACACATTCATTTATGCTGATGACATTATACT  3750
              ......3610......3620......3630......3640......3650......3660......3670......3680......3690......3700......3710......3720......3730......3740......3750

              ******************************************************************************************************************************************************
TEfam_I_Ele2  AATTACTTCGGACAAATCTGAAACAAAAAATAGAACCAAACTACAAAAAGCACTCAATAAACTTCATCATTGGTGCTGCTTGACAGGTCATGATCTATCAACAACAAAAAGTAAAATTCTACATATATGCAATAAATCACACAAACGAAC  3887
TESeeker_I    AATTACTTCGGACAAATCTGAAACAAAAAATAGAACCAAACTACAAAAAGCACTCAATAAACTTCATCATTGGTGCTGCTTGACAGGTCATGATCTATCAACAACAAAAAGTAAAATTCTACATATATGCAATAAATCACACAAACGAAC  3900
              ......3760......3770......3780......3790......3800......3810......3820......3830......3840......3850......3860......3870......3880......3890......3900

              ******************************************************************************************************************************************************
TEfam_I_Ele2  ACTTAAACCTATAAAAATTAAATACGATATAATTCCAAACGCAAGAGCAGTCAAAATTTTAGGAGTTATCATTGACTCAAGGTTAAAATTTCGACAACACCTTCTCTACGTTAAAAAAATGATCAAAAGCAGATTAAATATTCTGCATAT  4037
TESeeker_I    ACTTAAACCTATAAAAATTAAATACGATATAATTCCAAACGCAAGAGCAGTCAAAATTTTAGGAGTTATCATTGACTCAAGGTTAAAATTTCGACAACACCTTCTCTACGTTAAAAAAATGATCAAAAGCAGATTAAATATTCTGCATAT  4050
              ......3910......3920......3930......3940......3950......3960......3970......3980......3990......4000......4010......4020......4030......4040......4050

              ******************************************************************************************************************************************************
TEfam_I_Ele2  GTTAGGTTGTGGAAATAAGAGATCGGCAAGACGCACACTTCTCACTATTTTTAACAGTTGGTTTGTTCCAAAATTATTGTACGGCATTGAATTCATTACCATGGGCGATGCCAACTTTGGCTGCAAGCTAGCCCCAATGTACCACTCTGC  4187
TESeeker_I    GTTAGGTTGTGGAAATAAGAGATCGGCAAGACGCACACTTCTCACTATTTTTAACAGTTGGTTTGTTCCAAAATTATTGTACGGCATTGAATTCATTACCATGGGCGATGCCAACTTTGGCTGCAAGCTAGCCCCAATGTACCACTCTGC  4200
              ......4060......4070......4080......4090......4100......4110......4120......4130......4140......4150......4160......4170......4180......4190......4200

              ******************************************************************************************************************************************************
TEfam_I_Ele2  TCTGAAAAAAATTACAGGTGCTTTTATCACTAGCCCAAACACCGCAGTACTTTGTGAAAGTGGACAATTACCTCTCGACCATGTTGTAGTCCTTAAAATAGTAAACCTAGCTGGCAGACTCATTGAAAAAGGAATCGAAGCAAATTCTTT  4337
TESeeker_I    TCTGAAAAAAATTACAGGTGCTTTTATCACTAGCCCAAACACCGCAGTACTTTGTGAAAGTGGACAATTACCTCTCGACCATGTTGTAGTCCTTAAAATAGTAAACCTAGCTGGCAGACTCATTGAAAAAGGAATCGAAGCAAATTCTTT  4350
              ......4210......4220......4230......4240......4250......4260......4270......4280......4290......4300......4310......4320......4330......4340......4350

              ******************************************************************************************************************************************************
TEfam_I_Ele2  TGTAGATAGAGCCAACAAGGTGTTAAAACAACTAACAGGTCACAGCATGCCCAACATAGCTAGACTCACAAGCTGTTCGACTAGACCTTGGTACGACAAAATCCCCACAATCGACTGGACATTGCAAAAACTAAGCAATCACGAACAACA  4487
TESeeker_I    TGTAGATAGAGCCAACAAGGTGTTAAAACAACTAACAGGTCACAGCATGCCCAACATAGCTAGACTCACAAGCTGTTCGACTAGACCTTGGTACGACAAAATCCCCACAATCGACTGGACATTGCAAAAACTAAGCAATCACGAACAACA  4500
              ......4360......4370......4380......4390......4400......4410......4420......4430......4440......4450......4460......4470......4480......4490......4500

              ******************************************************************************************************************************************************
TEfam_I_Ele2  AATAGTACAAAACTATCAAATGCTCATCGCCAACAAATATAAAATATATAACAAAATTTATACTGATGGCTCGGTCTACAACAGTTCTGCGGGCTGTGGCATAACATCGCCAAATGCTCGCTGTAGCATTAAGCTTCCGGAGAACACATC  4637
TESeeker_I    AATAGTACAAAACTATCAAATGCTCATCGCCAACAAATATAAAATATATAACAAAATTTATACTGATGGCTCGGTCTACAACAGTTCTGCGGGCTGTGGCATAACATCGCCAAATGCTCGCTGTAGCATTAAGCTTCCGGAGAACACATC  4650
              ......4510......4520......4530......4540......4550......4560......4570......4580......4590......4600......4610......4620......4630......4640......4650

              ******************************************************************************************************************************************************
TEfam_I_Ele2  AATATTCTCAGCAGAGGCTATTGCTATTATGATAGCAACTGAAGAAGCGACCGTAAATGACAGACCTAACGTTATTTTCACAGATAGCGCTAGTGTTCTGAAGGCATTAGAGAAAGGATCCTTACGAAACCCCTACATACAAACTATCGA  4787
TESeeker_I    AATATTCTCAGCAGAGGCTATTGCTATTATGATAGCAACTGAAGAAGCGACCGTAAATGACAGACCTAACGTTATTTTCACAGATAGCGCTAGTGTTCTGAAGGCATTAGAGAAAGGATCCTTACGAAACCCCTACATACAAACTATCGA  4800
              ......4660......4670......4680......4690......4700......4710......4720......4730......4740......4750......4760......4770......4780......4790......4800
```

```
              ******************************************************************************************************************************************************
TEfam_I_Ele2  ATCACTATCAAAAGACAGGTCAATCGAATTCTGTTGGATACCAGGGCATAAAGGAATCGCAGGAAACGAAGTAGCTGACCGTCTTGCGAATGAAGGTAGGATGATCAAAGATCATGTAGATGGAACTATCTCTAGAAGCGATGCATCCTT  4937
TESeeker_I    ATCACTATCAAAAGACAGGTCAATCGAATTCTGTTGGATACCAGGGCATAAAGGAATCGCAGGAAACGAAGTAGCTGACCGTCTTGCGAATGAAGGTAGGATGATCAAAGATCATGTAGATGGAACTATCTCTAGAAGCGATGCATCCTT  4950
              ......4810......4820......4830......4840......4850......4860......4870......4880......4890......4900......4910......4920......4930......4940......4950

              ******************************************************************************************************************************************************
TEfam_I_Ele2  GTGGTGGAAAGGGGTAATAAATCAGAGCTGGCAAAACATTTGGAACAAATCACCCATTACCAACCTTAAAAAAATAAAACAAACCACGGCACCTTGGAACGATCCACTAGATCACAGTGATCAAAGAGTGGTAACAAGGTTGAGAATTGG  5087
TESeeker_I    GTGGTGGAAAGGGGTAATAAATCAGAGCTGGCAAAACATTTGGAACAAATCACCCATTACCAACCTTAAAAAAATAAAACAAACCACGGCACCTTGGAACGATCCACTAGATCACAGTGATCAAAGAGTGGTAACAAGGTTGAGAATTGG  5100
              ......4960......4970......4980......4990......5000......5010......5020......5030......5040......5050......5060......5070......5080......5090......5100

              ******************************************************************************************************************************************************
TEfam_I_Ele2  ACACACCCGACTTACACATACATACAAACTAAAAAAGGAATCACCGTTAATCTGCCCATTTTGTGGGTGTGATACAACGGTGGAACACATTTTAATTAATTGTTTTGGATATTCCGCAGAAAGGCAGAAACACAGACTTGGAGATCATTT  5237
TESeeker_I    ACACACCCGACTTACACATACATACAAACTAAAAAAGGAATCACCGTTAATCTGCCCATTTTGTGGGTGTGATACAACGGTGGAACACATTTTAATTAATTGTTTTGGATATTCCGCAGAAAGGCAGAAACACAGACTTGGAGATCATTT  5250
              ......5110......5120......5130......5140......5150......5160......5170......5180......5190......5200......5210......5220......5230......5240......5250

              *********************************************************************************.********************************************************************
TEfam_I_Ele2  GGATATTGTACTTTCCAACAATTACAAGGAAATTGAAAAACTTTTGAATTTTTTAAAGGAAACCAATCTCTACAAACAAGTGTAGCAAATATGGATAAAAATATTTATAAAAACATAATTGAAATGAAAAAAGCACTAGAATTAACAAAC  5387
TESeeker_I    GGATATTGTACTTTCCAACAATTACAAGGAAATTGAAAAACTTTTGAATTTTTTAAAGGAAACCAATCTCTACAAACAAGTATAGCAAATATGGATAAAAATATTTATAAAAACATAATTGAAATGAAAAAAGCACTAGAATTAACAAAC  5400
              ......5260......5270......5280......5290......5300......5310......5320......5330......5340......5350......5360......5370......5380......5390......5400

              ******************************************** *  ******************************************************************************************************
TEfam_I_Ele2  ATTTTCAACTTTCACCAGTACCATAATAACTCTTGTAATTTTTTTTTCTCTTTTTCTTGAACTTTTAATTTATTCTTTTTTTTGTATCTCAAATAGTATTAGCGATTTCTGAATTTTAAATTACGAGAGGCGAATGCTAAAAAGCCTCGT  5537
TESeeker_I    ATTTTCAACTTTCACCAGTACCATAATAACTCTTGTAATTTTTTCT--TCTTTTTCTTGAACTTTTAATTTATTCTTTTTTTTGTATCTCAAATAGTATTAGCGATTTCTGAATTTTAAATTACGAGAGGCGAATGCTAAAAAGCCTCGT  5548
              ......5410......5420......5430......5440......5450......5460......5470......5480......5490......5500......5510......5520......5530......5540......5550

              ************************
TEfam_I_Ele2  AAAAAAATAAAACAACAACAACAA----------------  5561
TESeeker_I    AAAAAAATAAAACAACAACAACAACCTTTACCGTCTTTCG  5588
              ......5560......5570......5580......5590
```

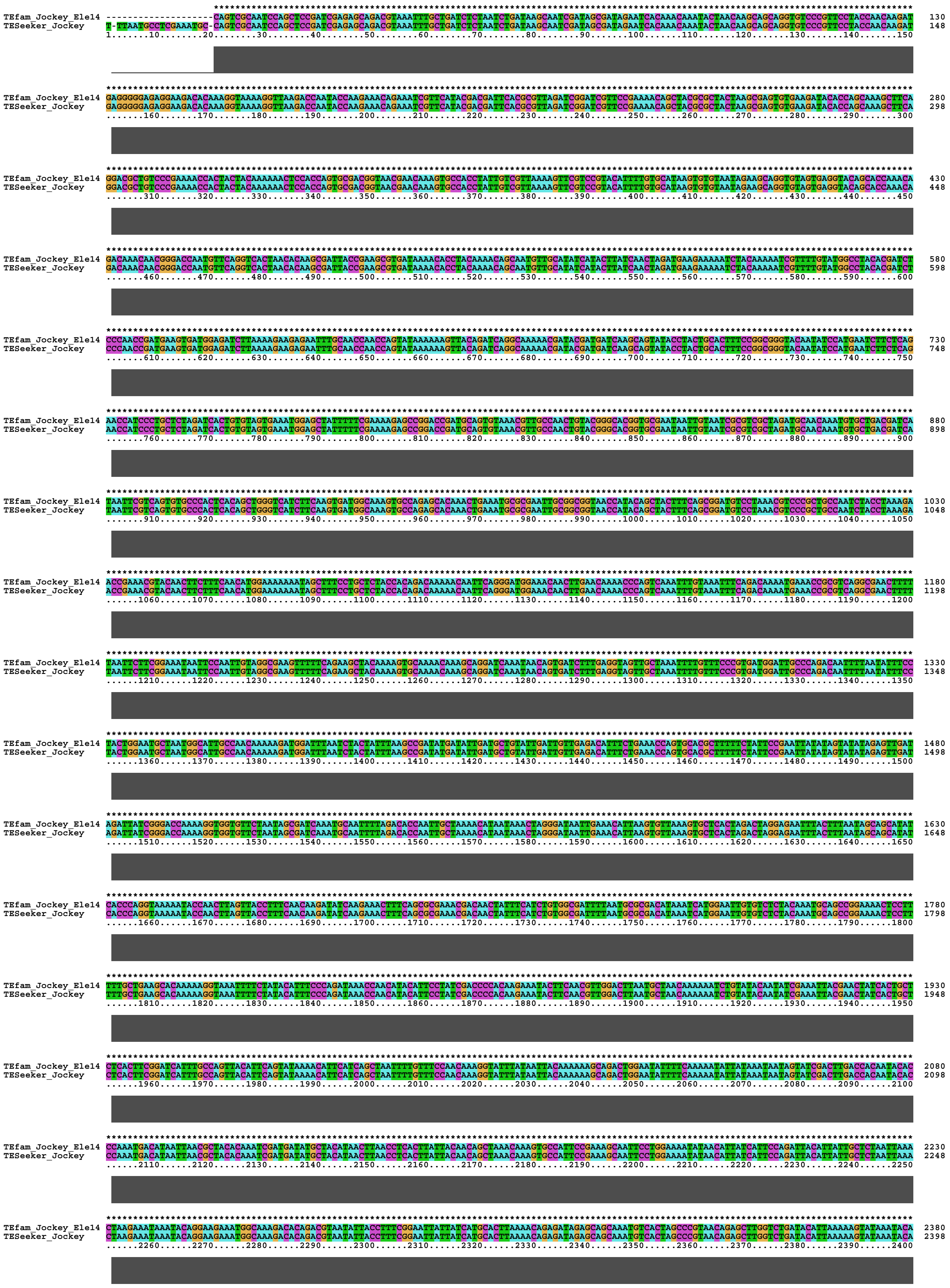

TEfam_Jockey_Ele14
TESeeker_Jockey
130
148
280
298
430
448
580
598
730
748
880
898
1030
1048
1180
1198
1330
1348
1480
1498
1630
1648
1780
1798
1930
1948
2080
2098
2230
2248
2380
2398

```
                   ******************************************************************************************************************************************************
TEfam_Jockey_Ele14 CAACAAAATAATACAAATAACCTATTCAAATTTGTAAAAATAGTTAAAAACAAGCAACATACTATTCAAACGCTAAAGCTAGGAAACAACATCTTCCTAACAGCCACTGAAAAATGTAACGCCCTCAAAACACATTTTGAAAACTCTCAT 2530
TESeeker_Jockey    CAACAAAATAATACAAATAACCTATTCAAATTTGTAAAAATAGTTAAAAACAAGCAACATACTATTCAAACGCTAAAGCTAGGAAACAACATCTTCCTAACAGCCACTGAAAAATGTAACGCCCTCAAAACACATTTTGAAAACTCTCAT 2548
                   ......2410......2420......2430......2440......2450......2460......2470......2480......2490......2500......2510......2520......2530......2540......2550

                   ******************************************************************************************************************************************************
TEfam_Jockey_Ele14 TACTTAACTTACAACACAAATAGTCCAATGGAAAGGAAAGTTACAAAAAACAATAGAATTTTCTTTGCGAGAAACCAAAATCACTCCTTGGATCCAGGTTACTTGGTTAAACCAAAATTACTCATGAACATAATCAAAAATCTTAAAAAT 2680
TESeeker_Jockey    TACTTAACTTACAACACAAATAGTCCAATGGAAAGGAAAGTTACAAAAAACAATAGAATTTTCTTTGCGAGAAACCAAAATCACTCCTTGGATCCAGGTTACTTGGTTAAACCAAAATTACTCATGAACATAATCAAAAATCTTAAAAAT 2698
                   ......2560......2570......2580......2590......2600......2610......2620......2630......2640......2650......2660......2670......2680......2690......2700

                   ******************************************************************************************************************************************************
TEfam_Jockey_Ele14 AAAAAATCAGCAGGCTGTGACGAAATTAACAACACAGAGATTAAAATGCTTCCTAAAAAGGCAGTACTCTACCTCAAAATAATATTTGATAGTTGTCTTAAACTAGGATACTTTCCTTCACAATGGAAAAAAGCAAAAATAGTGGCTATT 2830
TESeeker_Jockey    AAAAAATCAGCAGGCTGTGACGAAATTAACAACACAGAGATTAAAATGCTTCCTAAAAAGGCAGTACTCTACCTCAAAATAATATTTGATAGTTGTCTTAAACTAGGATACTTTCCTTCACAATGGAAAAAAGCAAAAATAGTGGCTATT 2848
                   ......2710......2720......2730......2740......2750......2760......2770......2780......2790......2800......2810......2820......2830......2840......2850

                   ******************************************************************************************************************************************************
TEfam_Jockey_Ele14 CCAAAACCCGGAAAAGATCATTCTAATCCTGGAAACTACAGACCAATATCCCTTCTGAGTTGCATAGGTAAACTTTTTGAGAAAATTATTGCACGCAATATACGTTCTCATACAGACATTAACAATATCATCCCACAGTCACAATTTGGT 2980
TESeeker_Jockey    CCAAAACCCGGAAAAGATCATTCTAATCCTGGAAACTACAGACCAATATCCCTTCTGAGTTGCATAGGTAAACTTTTTGAGAAAATTATTGCACGCAATATACGTTCTCATACAGACATTAACAATATCATCCCACAGTCACAATTTGGT 2998
                   ......2860......2870......2880......2890......2900......2910......2920......2930......2940......2950......2960......2970......2980......2990......3000

                   ******************************************************************************************************************************************************
TEfam_Jockey_Ele14 TTTCAACCCGAAAAATCAACAACTCATCAGATACATAGACTCAAAAATTATATTATAGAAAATCGGAATAGAAAAAGATCTACTGGTTTAGTTTTGTTAGATACTGAAAAAGCTTTCGATTCCATTTGGCATGACGGCCTGTTATATAAA 3130
TESeeker_Jockey    TTTCAACCCGAAAAATCAACAACTCATCAGATACATAGACTCAAAAATTATATTATAGAAAATCGGAATAGAAAAAGATCTACTGGTTTAGTTTTGTTAGATACTGAAAAAGCTTTCGATTCCATTTGGCATGACGGCCTGTTATATAAA 3148
                   ......3010......3020......3030......3040......3050......3060......3070......3080......3090......3100......3110......3120......3130......3140......3150

                   ******************************************************************************************************************************************************
TEfam_Jockey_Ele14 TTAATTCAATATAATTTTCCTACGTATATTATAAAAATAATTCAGTCCTTTCTCAGCAATCGAAAAAGTTCAGTTCATATTGGGCACACTAAATCAAATTTCTTTAAACCCATAGCGGGAGTACCACAAGGTAGTATATTATCGCCAATA 3280
TESeeker_Jockey    TTAATTCAATATAATTTTCCTACGTATATTATAAAAATAATTCAGTCCTTTCTCAGCAATCGAAAAAGTTCAGTTCATATTGGGCACACTAAATCAAATTTCTTTAAACCCATAGCGGGAGTACCACAAGGTAGTATATTATCGCCAATA 3298
                   ......3160......3170......3180......3190......3200......3210......3220......3230......3240......3250......3260......3270......3280......3290......3300

                   **********************************************************************************************.*******************************************************
TEfam_Jockey_Ele14 CTATTTAATATATTTATTTCTGACATTCCCTCTACAAACAACTGTACAAATTATCTATATGCAGACGACCTAGCAATCGCGGCTACGGCCAGAAATCCGAGCTCAATAATCAAAGCCTTAAATGGCACATTAAAAGCATACGCCAAGTTC 3430
TESeeker_Jockey    CTATTTAATATATTTATTTCTGACATTCCCTCTACAAACAACTGTACAAATTATCTATATGCAGACGACCTAGCAATCGCGGCTACGGCCAGAAGTCCGAGCTCAATAATCAAAGCCTTAAATGGCACATTAAAAGCATACGCCAAGTTC 3448
                   ......3310......3320......3330......3340......3350......3360......3370......3380......3390......3400......3410......3420......3430......3440......3450

                   **************************************:***************************************************************************************************************
TEfam_Jockey_Ele14 TGCAATAGATGGAAGCTTAAAACAAACGCAAATAAATCAGAGGCAATCTTCTTCACGAGAAACACTAGTTTACGCAAATTACCTAGTAATGAAGTTAGCTTTATGCAAGGAAACATTACATGGAAAGATCAAGTTAAATACCTTGGAGTA 3580
TESeeker_Jockey    TGCAATAGATGGAAGCTTAAAACAAACGCAAATAAATCTGAGGCAATCTTCTTCACGAGAAACACTAGTTTACGCAAATTACCTAGTAATGAAGTTAGCTTTATGCAAGGAAACATTACATGGAAAGATCAAGTTAAATACCTTGGAGTA 3598
                   ......3460......3470......3480......3490......3500......3510......3520......3530......3540......3550......3560......3570......3580......3590......3600

                   *****************************************************************************.************************************************************************
TEfam_Jockey_Ele14 CATTTAGAAAAACGCATGACATTTAAAACCCACATAGAAAAACAATTAGAAAAAGTAGACAAATTACTAAAAATTTTGTATCCTTTCCTAAATAGGAAATCAAAACTAAATTTGCAAAACAAAAAATTAGTTTACACGGCTTATATACGC 3730
TESeeker_Jockey    CATTTAGAAAAACGCATGACATTTAAAACCCACATAGAAAAACAATTAGAAAAAGTAGACAAATTACTAAAAATTTTATATCCTTTCCTAAATAGGAAATCAAAACTAAATTTGCAAAACAAAAAATTAGTTTACACGGCTTATATACGC 3748
                   ......3610......3620......3630......3640......3650......3660......3670......3680......3690......3700......3710......3720......3730......3740......3750

                   ******************************************************************************************************************************************************
TEfam_Jockey_Ele14 CCGATTCTGACGTACGCAGCGCCGGCATGGATTAGTTGTGCGTACTCTCATAGAAAAAAACTTCAAGTTAGACAAAACAAAATATTAAAAATGATCATGAACTTACCACCTTGGCATAACACAAACGATTTACACAAAAGAAGTAATTTA 3880
TESeeker_Jockey    CCGATTCTGACGTACGCAGCGCCGGCATGGATTAGTTGTGCGTACTCTCATAGAAAAAAACTTCAAGTTAGACAAAACAAAATATTAAAAATGATCATGAACTTACCACCTTGGCATAACACAAACGATTTACACAAAAGAAGTAATTTA 3898
                   ......3760......3770......3780......3790......3800......3810......3820......3830......3840......3850......3860......3870......3880......3890......3900

                   *******************************************************************************************************************:**********************************
TEfam_Jockey_Ele14 GTAAAAATGGAAGAATTTATAGGAAATTTAAATGTAAATTACGCAAACAAATGTCGCCAGAGCTCAATTAGCATAATTAATCAACTAGTTGAACAACGTATCTAAGCATAAAATAAGAGTTTATAATTTTCTGTACAAAGCTCTCCTCTT 4030
TESeeker_Jockey    GTAAAAATGGAAGAATTTATAGGAAATTTAAATGTAAATTACGCAAACAAATGTCGCCAGAGCTCAATTAGCATAATTAATCAACTAGTTGAACAACGTATCTAAGCATAAAATATGAGTTTATAATTTTCTGTACAAAGCTCTCCTCTT 4048
                   ......3910......3920......3930......3940......3950......3960......3970......3980......3990......4000......4010......4020......4030......4040......4050

                   *************************************************************************************************************************************.****************
TEfam_Jockey_Ele14 CTCTATCTAGTTAAGGATTAGGTCTAGCAAATGTAGGTTAAGATATAGGTTAGATATATATGTTTTTAGGTTAAGTCTAAAATTGCATGGGTTTTTTTTTGCACTTTTCCCATATACTCAGATTTAAAATCAGAAAACAACATATATTCT 4180
TESeeker_Jockey    CTCTATCTAGTTAAGGATTAGGTCTAGCAAATGTAGGTTAAGATATAGGTTAGATATATATGTTTTTAGGTTAAGTCTAAAATTGCATGGGTTTTTTTTTGCACTTTTCCCATATACTCAGATTTAAAATCAGCAAACAACATATATTCT 4198
                   ......4060......4070......4080......4090......4100......4110......4120......4130......4140......4150......4160......4170......4180......4190......4200

                   ****************************************************************************************************************************************
TEfam_Jockey_Ele14 ACTCAGCTATGCTATAAAAATTCCCGATTATTGAGGAGAGCACATTGCGTACCCAATGTTAAAGAACGTTCGATTCTATATGTATTTTTTTTTCTTTATATCCACAATAAACATCTCTACTACTACTACTACTACT---- 4316
TESeeker_Jockey    ACTCAGCTATGCTATAAAAATTCCCGATTATTGAGGAGAGCACATTGCGTACCCAATGTTAAAGAACGTTCGATTCTATATGTATTTTTTTTTCTTTATATCCACAATAAACATCTCTACTACTACTACTACTACTAATA 4338
                   ......4210......4220......4230......4240......4250......4260......4270......4280......4290......4300......4310......4320......4330......4340
```

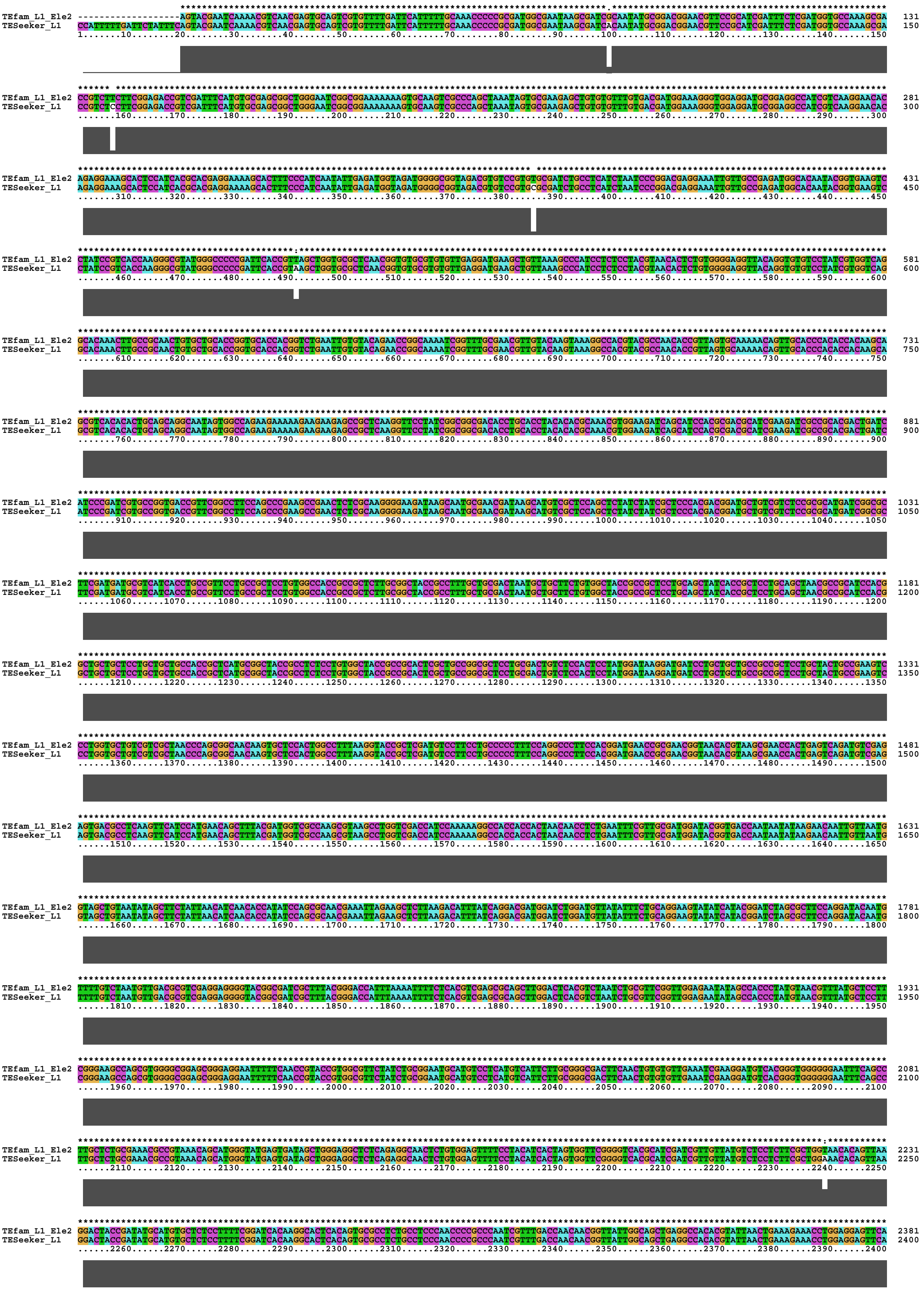
TEfam_L1_Ele2
TESeeker_L1

```
TEfam_L1_Ele2  GATGCAAGTGGAATAACTGGACTAAACAGCGTCGAAACTACGGCACGTGGATCTCGTGGTGGGTGGAGTTCGCGAAGCCTAAAATAAAATCATTTTTCCGCTGGAAAACAAATGAGAGATTCCGAAGTTTCCACTTGCACCACGAACTCC  2531
TESeeker_L1    GATGCAAGTGGAATAACTGGACTAAACAGCGTCGAAACTACGGCACGTGGATCTCGTGGTGGGTGGAGTTCGCGAAGCCTAAAATAAAATCATTTTTCCGCTGGAAAACAAATGAGAGATTCCGAAGTTTCCACTTGCACCACGAACTCC  2550
               ......2410......2420......2430......2440......2450......2460......2470......2480......2490......2500......2510......2520......2530......2540......2550

TEfam_L1_Ele2  TCTACAGAGGGTTGAAGTCCTCATACGAGCGATATCTGTCTGACCCTAACGAGTTGACGAATATCAATCGTATAAAAGGTAAAATGCTTCTCCTTCAGAGACGTTTTTCCGAGGACTTTAAACGTATCAATGAAACCCGCGTATGTGGCG  2681
TESeeker_L1    TCTACAGAGGGTTGAAGTCCTCATACGAGCGATATCTGTCTGACCCTAACGAGTTGACGAATATCAATCGTATAAAAGGTAAAATGCTTCTCCTTCAGAGACGTTTTTCCGAGGACTTTAAACGTATCAATGAAACCCGCGTATGTGGCG  2700
               ......2560......2570......2580......2590......2600......2610......2620......2630......2640......2650......2660......2670......2680......2690......2700

TEfam_L1_Ele2  AAAACATCTCGACCTTCCAGCTTGAGGAAAGGAGGAGGAGGCGAACTGTGATCGAAAAACTAAATATTGAAGACGGGACATCCTTAACTGATAAAGACGCGATAAATGTTCACATTAGGAGCTTCTTTTCAGAGCTATACACCACAGACA  2831
TESeeker_L1    AAAACATCTCGACCTTCCAGCTTGAGGAAAGGAGGAGGAGGCGAACTGTGATCGAAAAACTAAATATTGAAGACGGGACATCCTTAACTGATAAAGACGCGATAAATGTTCACATTAGGAGCTTCTTTTCAGAGCTATACACCACAGACA  2850
               ......2710......2720......2730......2740......2750......2760......2770......2780......2790......2800......2810......2820......2830......2840......2850

TEfam_L1_Ele2  ACACAGACAACACACAAACAGACGACACATTTACATGCACACGCGTCATTCCTGAGGATTGTGAGATCAATAATGGTTGCATGGAGGAAATCACTTCTGGTGAGATCCTCTATGCGATCAAAAACTCACAGTCTCGAAAGTCCCCTGGCC  2981
TESeeker_L1    ACACAGACAACACACAAACAGACGACACATTTACATGCACACGCGTCATTCCTGAGGATTGTGAGATCAATAATGGTTGCATGGAGGAAATCACTTCTGGTGAGATCCTCTATGCGATCAAAAACTCACAGTCTCGAAAGTCCCCTGGCC  3000
               ......2860......2870......2880......2890......2900......2910......2920......2930......2940......2950......2960......2970......2980......2990......3000

TEfam_L1_Ele2  CAGATGGAATACCCAAGGAGTTTATCTCCGCGCGTTCGACGTCATCGAGCGCGAGCTTGGGCTCGTGCTCAATGAGGCACTCCGCGGAGAGATTCCTCGAAGCTTCGTCGACGGGGTCATAGTGCTGGTGAGGAAAAAGGGGGGTGGGG  3131
TESeeker_L1    CAGATGGAATACCCAAGGAGTTTATCTCCGCGCGTTCGACGTCATCGAGCGCGAGCTTGGGCTCGTGCTCAATGAGGCACTCCGCGGAGAGATTCCTCGAAGCTTCGTCGACGGGGTCATAGTGCTGGTGAGGAAAAAGGGGGGTGGGG  3150
               ......3010......3020......3030......3040......3050......3060......3070......3080......3090......3100......3110......3120......3130......3140......3150

TEfam_L1_Ele2  ATGCCATGTCTTCAATTCGACCTATATCACTCCTTAACACAGACTACAAGCTTTTGTCGAAGGTTATTAAACCCCGTCTCGATTGTATAATCGGGAAATGGGGGATAATCTCGACAGCGCAAAAGTGTTGCAACAAACCTTGCAACATTT  3281
TESeeker_L1    ATGCCATGTCTTCAATTCGACCTATATCACTCCTTAACACAGACTACAAGCTTTTGTCGAAGGTTATTAAACCCCGTCTCGATTGTATAATCGGGAAATGGGGGATAATCTCGACAGCGCAAAAGTGTTGCAACAAACCTTGCAACATTT  3300
               ......3160......3170......3180......3190......3200......3210......3220......3230......3240......3250......3260......3270......3280......3290......3300

TEfam_L1_Ele2  TTCAAGCTGTACTCTCTGTTAAGGAGAGGGTGGTTGATTTGAAGATGAAACGCAAGTGTGGTAAACTAGTCTCCTTTGATCTCTCGCAGGCGTTCGATCGCGTCGATAGAGGCTTCCTCTTTAATAACATGGTTTCTATGGGTTTCAATC  3431
TESeeker_L1    TTCAAGCTGTACTCTCTGTTAAGGAGAGGGTGGTTGATTTGAAGATGAAACGCAAGTGTGGTAAACTAGTCTCCTTTGATCTCTCGCAGGCGTTCGATCGCGTCGATAGAGGCTTCCTCTTTAATAACATGGTTTCTATGGGTTTCAATC  3450
               ......3310......3320......3330......3340......3350......3360......3370......3380......3390......3400......3410......3420......3430......3440......3450

TEfam_L1_Ele2  CTGGGCTGGTGGGGCTTTTGAGAAGGTTTGGTGATCAGTCCTCCTCCCGTATCCTAGTCAACGGATCCCTATCTCCCCCTATTTCCATCCGACGATCCGTTCGTCAGGGGGATCCACTTTCCATGCACCTTTTCATCCTATACCTTCATC  3581
TESeeker_L1    CTGGGCTGGTGGGGCTTTTGAGAAGGTTTGGTGATCAGTCCTCCTCCCGAATCCTAGTCAACGGATCCCTATCTCCCCCTATTTCCATCCGACGATCCGTTCGTCAGGGGGATCCACTTTCCATGCACCTTTTCATCCTATACCTTCATC  3600
               ......3460......3470......3480......3490......3500......3510......3520......3530......3540......3550......3560......3570......3580......3590......3600

TEfam_L1_Ele2  CCCTCATCACCAGACTAGAAGGCATATGCTGCGACCAGGATGACCTGGTCAATGCGTACGCCGACGACATCTCGGTGGTGACTACCTCATCCCAAAAAATCGAGTTGGTGCGTGAGGCTTTTGAAGCGTTTGGCAGAGTCTCCGGAGCCC  3731
TESeeker_L1    CCCTCATCACCAGACTAGAAGGCATATGCTGCGACCAGGATGACCTGGTCAATGCGTACGCCGACGACATCTCGGTGGTGACTACCTCATCCCAAAAAATCGAGTTGGTGCGTGAGGCTTTTGAAGCGTTTGGCAGAGTCTCCGGAGCCC  3750
               ......3610......3620......3630......3640......3650......3660......3670......3680......3690......3700......3710......3720......3730......3740......3750

TEfam_L1_Ele2  GCCTCAACGTCGACAAAACCATCGCGCTCGACGTGGGATACACCACATCAAATGCCATTCAGGTCCCTTGGCTTCGTACCGTGGAGAGGCTTAGGCTCCTCGGTATATTGTTTACCAACAATGTACGAGAAGCTATGAGCCTCAACTGGG  3881
TESeeker_L1    GCCTCAACGTCGACAAAACCATCGCGCTCGACGTGGGATACACCACATCAAATGCCATTCAGGTCCCTTGGCTTCGTACCGTGGAGAGGCTAAGGCTCCTCGGTATATTGTTTACCAACAATGTACGAGAAGCTATGAGCCTCAACTGGG  3900
               ......3760......3770......3780......3790......3800......3810......3820......3830......3840......3850......3860......3870......3880......3890......3900

TEfam_L1_Ele2  ACCTGTTGATCCACCATTTCCGACAGCTGGTGTGGCTTCATCGCGTGAGGGACTTAAACGTGGTGCAGAAGGTTGTTCTGCTGAACACCTTCCTACTCCCCAAGCTGTGGTTTGTTGCATCGGTTTGTGGCGCCCGTGCAATGGACATAG  4031
TESeeker_L1    ACCTGTTGATCCACCATTTCCGACAGCTGGTGTGGCTTCATCGCGTGAGGGACTTAAACGTGGTGCAGAAGGTTGTTCGCTGAACACCTTCCTACTCCCCAAGCTGTGGTTTGTTGCATCGGTTTGTGGCGCCCGTGCAATGGACATAG  4050
               ......3910......3920......3930......3940......3950......3960......3970......3980......3990......4000......4010......4020......4030......4040......4050

TEfam_L1_Ele2  CGAAGGTGACCTGCACCGTGAACACGTTCCTCTGGGATGGATCCGGAGGCTTCCGAGTCCCACTGCAGCAACTGGCGCTGCCTCGCAACCGTGGTGGGCTTAACCTTCACCTTCCTGCCATCATGGCCAAGGCGCTGTTGACGAACCGGT  4181
TESeeker_L1    CGAAGGTGACCTGCACCGTGAACACGTTCCTCTGGGATGGATCCGGAGGCTTCCGAGTCCCACTGCAGCAACTGGCACTGCCTCGCAACCGTGGTGGGCTTAACCTTCACCTTCCTGCCATCATGGCCAAGGCGCTGTTGACGAACCGGT  4200
               ......4060......4070......4080......4090......4100......4110......4120......4130......4140......4150......4160......4170......4180......4190......4200

TEfam_L1_Ele2  ATGTTACGGAACAGGACTGTCTACGAGTCGGAGGTCAGCACATTTCTTGTGCTGGGAATCCTCCAAACATCGCCGCTGTTTCGTCAACGTACCCGTGTCTGCGTATCGTCATACAGCAACTTGGCTACCAACCCCCATCAGCCACCATCA  4331
TESeeker_L1    ATGTTACGGAACAGGACTGTCTACGAGTCGGAGGTCAGCACATTTCTTGTGCTGGGAATCCTCCAAACATCGCCGCTGTTTCGTCAACGTACCCGTGTCTGCGTATCGTCATACAGCAACTTGGCTACCAACCACCATCAGCCACCATCA  4350
               ......4210......4220......4230......4240......4250......4260......4270......4280......4290......4300......4310......4320......4330......4340......4350

TEfam_L1_Ele2  CGACACGCTGGATTCGCCAAACAATGACGGAAGTGCTTCTTGAGCCGAAGGTAGCCTTGGAAAATCCGTCAGTGAATTGGCGGCTACTCTGGCGCAATATTCATCGTTCCTGTCTATCGTCCCTTCAACGCAGCACGCTCTTCTTGCTGG  4481
TESeeker_L1    CGACACGCTGGATTCGCCAAACAATGACGGAAGTGCTTCTTGAGCCGAAGGTAGCCTTGGAAAATCCGTCAGTGAATTGGCGGCTACTCTGGCGCAATATTCATCGTTCCTGTCTATCGTCCCTTCAACGCAGCACGCTCTTCTTGCTGG  4500
               ......4360......4370......4380......4390......4400......4410......4420......4430......4440......4450......4460......4470......4480......4490......4500

TEfam_L1_Ele2  TAAACGGCAAGATCAGCCATGGAGAGCTGCTGCATCGAATGAACCGCGTCCCATCTCCGTCATGTTCGTTTTGTTCTGCAATAGACACCCTGGAACACAAATTCGCTGGCTGCAGAAGAGTTGCTGATGCTTGGCAGATTCTGCACCAGC  4631
TESeeker_L1    TAAACGGCAAGATCAGCCATGGAGAGCTGCTGCATCGAATGAACCGCGTCCCATCTCCGTCATGTTCGTTTTGTTCTGCAATAGACACCCTGGAACACAAATTCGCTGGCTGCAGAAGAGTTGCTGATGCTTGGCAGATTCTGCACCAGC  4650
               ......4510......4520......4530......4540......4550......4560......4570......4580......4590......4600......4610......4620......4630......4640......4650

TEfam_L1_Ele2  GAATTAGCGTTGTAATTTCAGGGTGTCCGTCTTCGTCAACGTTATTGTTCGGTTTGTTGCGTTTGCCTGTACTAAACGGCATTAGTGCAGGTAAACGCAGCCATATCCTAAGTTTGTTGCGAACTATGTCATCCACATCATTGGAAACA  4781
TESeeker_L1    GAATTAGCGTTGTAATTTCAGGGTGTCCGTCTTCGTCAACGTTATTGTTCGGTTTGTTGCGTTTGCCTGTACTAAACGGCATTAATGCAGGTAAACGCAGCCATATCCTAAGTTTGTTGCGAACTATGTCATCCACATCATTGGAAACA  4800
               ......4660......4670......4680......4690......4700......4710......4720......4730......4740......4750......4760......4770......4780......4790......4800
```

```
                  *****************************************************************************************
TEfam_L1_Ele2 ATGATGCTGTGATTGACATAGAAGTTCTGCGTTGTTCATTGTAAAAATTAATGTTAAATAGAACCTAAGCACGTTTTTAATAAAACATT------------  4870
TESeeker_L1   ATGATGCTGTGATTGACATAGAAGTTCTGCGTTGTTCATTGTAAAAATTAATGTTAAATAGAACCTAAGCACGTTTTTAATAAAACATTTATAAAAAAAAA  4901
              ......4810......4820......4830......4840......4850......4860......4870......4880......4890......4900.
```

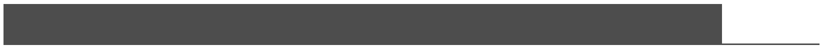

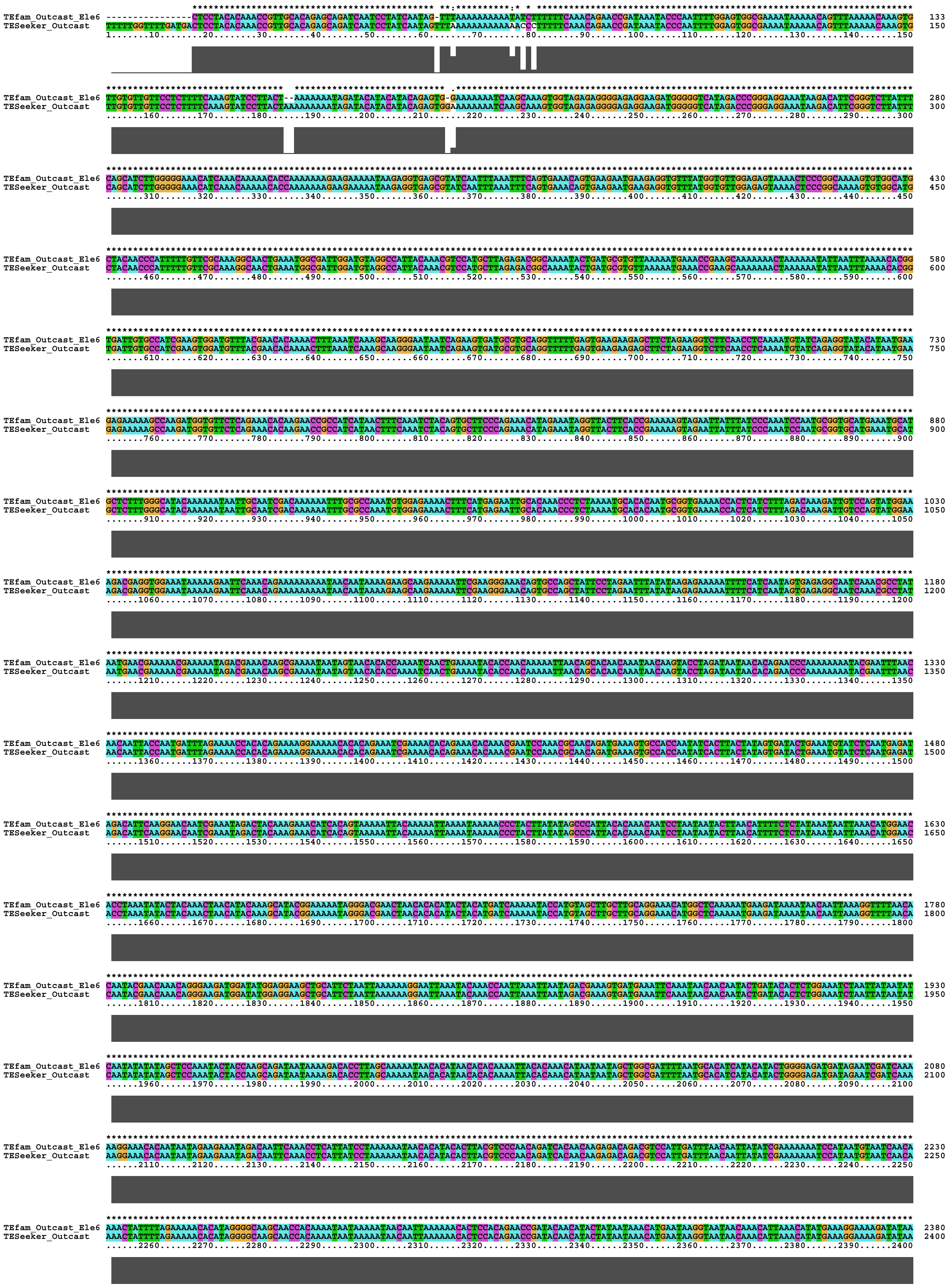
TEfam_Outcast_Ele6
TESeeker_Outcast

```
                   ******************************************************************************************************************************************************
TEfam_Outcast_Ele6 TAAACATAAAACATTTCACAAAGAAAGTATACAAAATCATACAAAATAGTAAACAAAAAATCAATTTTACACTAAAATCATGGTGGAATAACGAAATAAAAAAGGCCCTGCAAGACAAAAATATAGCAAGACAAACATTCAACAGTACAA  2530
TESeeker_Outcast   TAAACATAAAACATTTCACAAAGAAAGTATACAAAATCATACAAAATAGTAAACAAAAAATCAATTTTACACTAAAATCATGGTGGAATAACGAAATAAAAAAGGCCCTGCAAGACAAAAATATAGCAAGACAAACATTCAACAGTACAA  2550
                   ......2410......2420......2430......2440......2450......2460......2470......2480......2490......2500......2510......2520......2530......2540......2550

                   ******************************************************************************************************************************************************
TEfam_Outcast_Ele6 AACTTATTGAACATGCCATAGAATTCCGCAAAAGGACAGCAATCTTCAAACTTAAAATAAAACAAGCAAAAGAAAAACAAACGGAGAGAAACATGGAAAAAATAAACAAAGACACAAGTAGTAAAGAACTATGGAATCTGTTAGGAAATA  2680
TESeeker_Outcast   AACTTATTGAACATGCCATAGAATTCCGCAAAAGGACAGCAATCTTCAAACTTAAAATAAAACAAGCAAAAGAAAAACAAACGGAGAGAAACATGGAAAAAATAAACAAAGACACAAGTAGTAAAGAACTATGGAATCTGTTAGGAAATA  2700
                   ......2560......2570......2580......2590......2600......2610......2620......2630......2640......2650......2660......2670......2680......2690......2700

                   ******************************************************************************************************************************************************
TEfam_Outcast_Ele6 TTAGCAACATCAATACCACCAATAAAGAGTCAAACCTAATTCACAATGAAGAGACCTATGCTAAGGAATTTATGAATTTAAATTTCACAAACAATAAAAAATCCAAATTTAGAACTTTTTTCAGTACAAATCTACCGATAGATACGGAAC  2830
TESeeker_Outcast   TTAGCAACATCAATACCACCAATAAAGAGTCAAACCTAATTCACAATGAAGAGACCTATGCTAAGGAATTTATGAATTTAAATTTCACAAACAATAAAAAATCCAAATTTAGAACTTTTTTCAGTACAAATCTACCGATAGATACGGAAC  2850
                   ......2710......2720......2730......2740......2750......2760......2770......2780......2790......2800......2810......2820......2830......2840......2850

                   ******************************************************************************************************************************************************
TEfam_Outcast_Ele6 TTATAAATTCAGATATATGGAAAAACATACTGAAACAAAAAAAAAATACAACACCCGGTTTAGACAAAATTACATACCAAATGCTAAGAAACATAAACGACGAATCACTAACACAAATCATAACAGACGGAAACAAAATGTGGGAGAATG  2980
TESeeker_Outcast   TTATAAATTCAGATATATGGAAAAACATACTGAAACAAAAAAAAAATACAACACCCGGTTTAGACAAAATTACATACCAAATGCTAAGAAACATAAACGACGAATCACTAACACAAATCATAACAGACGGAAACAAAATGTGGGAGAATG  3000
                   ......2860......2870......2880......2890......2900......2910......2920......2930......2940......2950......2960......2970......2980......2990......3000

                   ******************************************************************************************************************************************************
TEfam_Outcast_Ele6 GTAAAATAAACAAAGAACTAAAACAAATTAAAATAATAGCAATTCCTAAACCAAAGAAAAATATAAATGACCCCAACAACTATAGACCTATAGCACTTATACCAACAATAACCAAAGTCCTAAACTCAGCGGTATTATTAAAATTAAATA  3130
TESeeker_Outcast   GTAAAATAAACAAAGAACTAAAACAAATTAAAATAATAGCAATTCCTAAACCAAAGAAAAATATAAATGACCCCAACAACTATAGACCTATAGCACTTATACCAACAATAACCAAAGTCCTAAACTCAGCGGTATTATTAAAATTAAATA  3150
                   ......3010......3020......3030......3040......3050......3060......3070......3080......3090......3100......3110......3120......3130......3140......3150

                   ******************************************************************************************************************************************************
TEfam_Outcast_Ele6 AATATATTGAAACCAAAAATATACTCCCGGAAAAATCCTTTGGATTCAGAAACAAAAGAACCATAAACCAATGCATTAACTATTTCAATAACGAAATAAATCACAACCAAAGACTAAATAGAATAAGTGGAGCAATATTCATAGATTTGG  3280
TESeeker_Outcast   AATATATTGAAACCAAAAATATACTCCCGGAAAAATCCTTTGGATTCAGAAACAAAAGAACCATAAACCAATGCATTAACTATTTCAATAACGAAATAAATCACAACCAAAGACTAAATAGAATAAGTGGAGCAATATTCATAGATTTGG  3300
                   ......3160......3170......3180......3190......3200......3210......3220......3230......3240......3250......3260......3270......3280......3290......3300

                   ******************************************************************************************************************************************************
TEfam_Outcast_Ele6 AAAAAGCATATAATAATGTTTCAACAAAAATCCTCGTGGAACAAATGATAAAATCAGACATCCCACCTCAAATTATAAAATGGACCTACTCCTTTCTTAGAAATAGAACTTTAATAATAGAAAGCAATAAAAAAACATACAAAATGTTAG  3430
TESeeker_Outcast   AAAAAGCATATAATAATGTTTCAACAAAAATCCTCGTGGAACAAATGATAAAATCAGACATCCCACCTCAAATTATAAAATGGACCTACTCCTTTCTTAGAAATAGAACTTTAATAATAGAAAGCAATAAAAAAACATACAAAATGTTAG  3450
                   ......3310......3320......3330......3340......3350......3360......3370......3380......3390......3400......3410......3420......3430......3440......3450

                   ******************************************************************************************************************************************************
TEfam_Outcast_Ele6 TGACAGATGGACTACCACAAGGAGATGTTATGTCACCAACACTTTTTAACTTATATACCAAAGATATACATAAAGCTATAAACCAAACAAACACCAAAAATACTATAATACAGTACGCGGATGATTTCGTTATTCTCAGCAGTGGAGTAA  3580
TESeeker_Outcast   TGACAGATGGACTACCACAAGGAGATGTTATGTCACCAACACTTTTTAACTTATATACCAAAGATATACATAAAGCTATAAACCAAACAAACACCAAAAATACTATAATACAGTACGCGGATGATTTCGTTATTCTCAGCAGTGGAGTAA  3600
                   ......3460......3470......3480......3490......3500......3510......3520......3530......3540......3550......3560......3570......3580......3590......3600

                   ******************************************************************************************************************************************************
TEfam_Outcast_Ele6 ACGAAAGAGAGCTACAAAATAACCTACAAAACGCTTTAAATGCTTTCGCAATAGAAACAGAACAACTAAAATTTAACATAAACTCAAACAAAACAAAATTTATGATTTTCGGAAAATCACATCACACCATACAACTCAACATACACAATA  3730
TESeeker_Outcast   ACGAAAGAGAGCTACAAAATAACCTACAAAACGCTTTAAATGCTTTCGCAATAGAAACAGAACAACTAAAATTTAACATAAACTCAAACAAAACAAAATTTATGATTTTCGGAAAATCACATCACACCATACAACTCAACATACACAATA  3750
                   ......3610......3620......3630......3640......3650......3660......3670......3680......3690......3700......3710......3720......3730......3740......3750

                   ******************************************************************************************************************************************************
TEfam_Outcast_Ele6 ATAGCATAGAACACACAAACACATACAAATATCTAGGAACAGTAATAGATCCAAAACTCAATTTCCACAAACACATCGAACTACTACGTAACAAGGCTACAAACAGACTAAATTTCTTGAAAATTATAAGCACGCAAAAAAATAACATAA  3880
TESeeker_Outcast   ATAGCATAGAACACACAAACACATACAAATATCTAGGAACAGTAATAGATCCAAAACTCAATTTCCACAAACACATCGAACTACTACGTAACAAGGCTACAAACAGACTAAATTTCTTGAAAATTATAAGCACGCAAAAAAATAACATAA  3900
                   ......3760......3770......3780......3790......3800......3810......3820......3830......3840......3850......3860......3870......3880......3890......3900

                   ******************************************************************************************************************************************************
TEfam_Outcast_Ele6 ACCCCAAAAACAGCCTAAAAATATACCGGGCAACAATCAGAAACACTATGGAAACGGGAGCTTCATACACTCTCAATAGCAACAAAAACAAATACAAAACAATGAATTCAACTATCAACCAGGCACTAAGAAAAGCTACAGGTTGCACCA  4030
TESeeker_Outcast   ACCCCAAAAACAGCCTAAAAATATACCGGGCAACAATCAGAAACACTATGGAAACGGGAGCTTCATACACTCTCAATAGCAACAAAAACAAATACAAAACAATGAATTCAACTATCAACCAGGCACTAAGAAAAGCTACAGGTTGCACCA  4050
                   ......3910......3920......3930......3940......3950......3960......3970......3980......3990......4000......4010......4020......4030......4040......4050

                   ******************************************************************************************************************************************************
TEfam_Outcast_Ele6 AAACTACCCCCATAAATACTCTGCATGCCATTGCAGCAGAAATACCTTTCAATATCAGAAGTAGATTTATAGTACGTAAGGAACTAGCCAAAGATTTAGTCTACTCACCAATCATTAGACAACAACTACAAATACATAAAAGAACAAAAT  4180
TESeeker_Outcast   AAACTACCCCCATAAATACTCTGCATGCCATTGCAGCAGAAATACCTTTCAATATCAGAAGTAGATTTATAGTACGTAAGGAACTAGCCAAAGATTTAGTCTACTCACCAATCATTAGACAACAACTACAAATACATAAAAGAACAAAAT  4200
                   ......4060......4070......4080......4090......4100......4110......4120......4130......4140......4150......4160......4170......4180......4190......4200

                   ******************************************************************************************************************************************************
TEfam_Outcast_Ele6 ACAAAAAAAAGAAAAAAACAATACATGAAACAACGTACGAAAAAGACAATAAAATTCTAAAACAATTATACATTGCAAAAAACAACGATATAACCACACACATCAAAATAAATACAAAAATTAAAGACACGATAGAAACAAAAACAAAAA  4330
TESeeker_Outcast   ACAAAAAAAAGAAAAAAACAATACATGAAACAACGTACGAAAAAGACAATAAAATTCTAAAACAATTATACATTGCAAAAAACAACGATATAACCACACACATCAAAATAAATACAAAAATTAAAGACACGATAGAAACAAAAACAAAAA  4350
                   ......4210......4220......4230......4240......4250......4260......4270......4280......4290......4300......4310......4320......4330......4340......4350

                   ******************************************************************************************************************************************************
TEfam_Outcast_Ele6 CAAACACTCAAATCCTGAAACAAATTACCATTGAAAACATGAACAAGTACGAAAATAACAGACCCACCATCTACACTGATGGAAGCATTGCCGGAAATAAAGTAGGTATAGGGATATACATAAAAAACAAACCACAACATTACTACTATA  4480
TESeeker_Outcast   CAAACACTCAAATCCTGAAACAAATTACCATTGAAAACATGAACAAGTACGAAAATAACAGACCCACCATCTACACTGATGGAAGCATTGCCGGAAATAAAGTAGGTATAGGGATATACATAAAAAACAAACCACAACATTACTACTATA  4500
                   ......4360......4370......4380......4390......4400......4410......4420......4430......4440......4450......4460......4470......4480......4490......4500

                   ******************************************************************************************************************************************************
TEfam_Outcast_Ele6 GCTATAGATTAAAAAATTTCACCTCCATAACCACCGCCGAACTGATAGCAATCGAAAAGGCCTTACTTTTAGCACAAGAAAACAATATAGACAACCCAGTAATTTTCACAGACAGTCTAACATCATGTAACATTCTACAAAAAGCAATGA  4630
TESeeker_Outcast   GCTATAGATTAAAAAATTTCACCTCCATAACCACCGCCGAACTGATAGCAATCGAAAAGGCCTTACTTTTAGCACAAGAAAACAATATAGACAACCCAGTAATTTTCACAGACAGTCTAACATCATGTAACATTCTACAAAAAGCAATGA  4650
                   ......4510......4520......4530......4540......4550......4560......4570......4580......4590......4600......4610......4620......4630......4640......4650

                   ******************************************************************************************************************************************************
TEfam_Outcast_Ele6 CAGAAAACAAAATAGAGGAAATCTGTTACAATATTATAAAAACAGCAACAAATATTAAAGCAGATATTGTATGGATTCCATCACATGTAGGCATTGATGGAAATGATAGAGCAGATGAACTGGCAAAAAAAGGCACTTTAACAAATTGGT  4780
TESeeker_Outcast   CAGAAAACAAAATAGAGGAAATCTGTTACAATATTATAAAAACAGCAACAAATATTAAAGCAGATATTGTATGGATTCCATCACATGTAGGCATTGATGGAAATGATAGAGCAGATGAACTGGCAAAAAAAGGCACTTTAACAAATTGGT  4800
                   ......4660......4670......4680......4690......4700......4710......4720......4730......4740......4750......4760......4770......4780......4790......4800
```

```
                   ******************************************************************************************************************************************************
TEfam_Outcast_Ele6 TCTATAAGAACAAAATCAGATACACAGACGCTACCCAATATTACAAAAAAGAAATGGAAGAGGAAACAAAAAAATGGTACACAGACCTGGGCAGGAATAAAGGGAAAAAGTTTATGCAATTCCAAAATGTTTTCAAAACAGAACTATGGC  4930
TESeeker_Outcast   TCTATAAGAACAAAATCAGATACACAGACGCTACCCAATATTACAAAAAAGAAATGGAAGAGGAAACAAAAAAATGGTACACAGACCTGGGCAGGAATAAAGGGAAAAAGTTTATGCAATTCCAAAATGTTTTCAAAACAGAACTATGGC  4950
                   ......4810......4820......4830......4840......4850......4860......4870......4880......4890......4900......4910......4920......4930......4940......4950

                   ******************************************************************************************************************************************************
TEfam_Outcast_Ele6 ACAAACAGGTTAACCTGAACGGAAATGAAGTAAAAACAATTAACAAAATACTGGCAGGACATGACCTTTCCGAATTTTGGCTTCATAAAATGAAAATAGTAGAAAATGGAACCTGTGAGAAATGTCTAGTCCCAGAAACAGGACGACACA  5080
TESeeker_Outcast   ACAAACAGGTTAACCTGAACGGAAATGAAGTAAAAACAATTAACAAAATACTGGCAGGACATGACCTTTCCGAATTTTGGCTTCATAAAATGAAAATAGTAGAAAATGGAACCTGTGAGAAATGTCTAGTCCCAGAAACAGGACGACACA  5100
                   ......4960......4970......4980......4990......5000......5010......5020......5030......5040......5050......5060......5070......5080......5090......5100

                   ******************************************************************************************************************************************************
TEfam_Outcast_Ele6 AAATTTTTGAGTGTCAAAAATATAAAAGACATAAAGACATCTCCCTAGACACTTTGTACGAAAAATGGATGGAAACCAGTGGAAATACCTGCAAAAAAATTATAGAATTCATAAAAGAAAACAATATTACATTATAGTACTTCATCACAC  5230
TESeeker_Outcast   AAATTTTTGAGTGTCAAAAATATAAAAGACATAAAGACATCTCCCTAGACACTTTGTACGAAAAATGGATGGAAACCAGTGGAAATACCTGCAAAAAAATTATAGAATTCATAAAAGAAAACAATATTACATTATAGTACTTCATCACAC  5250
                   ......5110......5120......5130......5140......5150......5160......5170......5180......5190......5200......5210......5220......5230......5240......5250

                   *************************************** **************************************************************************************************************
TEfam_Outcast_Ele6 ATATCTTCAAATAAAAAGAAAAATTCAACAATTTTTTTTTACACATTTCCTATATCTTCAAAACTCACAACATAGAACCTGCACTCAACACTCCAGCAAAAATGATATAGCAATAGGATAAGTACTTACTATTTATTTATCTATTTATTA  5380
TESeeker_Outcast   ATATCTTCAAATAAAAAGAAAAATTCAACAATTTTTTTT-ACACATTTCCTATATCTTCAAAACTCACAACATAGAACCTGCACTCAACACTCCAGCAAAAATGATATAGCAATAGGATAAGTACTTACTATTTATTTATCTATTTATTA  5399
                   ......5260......5270......5280......5290......5300......5310......5320......5330......5340......5350......5360......5370......5380......5390......5400

                   ******************************************************************************************************************************************************
TEfam_Outcast_Ele6 TATTTATTACATCTATTGCATTTATTATATTTATTCTATTTATTATATTTATTATATTTATCATATTTATGTGTTCAAAGTATCAATTTTGTTCGTATAATGAAACGTTATTCCATGTTTTTTTTTCCTTGTTAGTCAATTATTTAAAAC  5530
TESeeker_Outcast   TATTTATTACATCTATTGCATTTATTATATTTATTCTATTTATTATATTTATTATATTTATCATATTTATGTGTTCAAAGTATCAATTTTGTTCGTATAATGAAACGTTATTCCATGTTTTTTTTTCCTTGTTAGTCAATTATTTAAAAC  5549
                   ......5410......5420......5430......5440......5450......5460......5470......5480......5490......5500......5510......5520......5530......5540......5550

                   ******************************************************************************************************************************************************
TEfam_Outcast_Ele6 TATTTATTCAATCATTTCTCAGCACCCTATTGATTTATTTATTTATTTATATACTGTTTTTGGCATGACTTATATATATACACAAAAAATCACCTAACTGCACATTCCAAATCCAAACACCACACCACAAAAAAAATAACAATAACAATA  5680
TESeeker_Outcast   TATTTATTCAATCATTTCTCAGCACCCTATTGATTTATTTATTTATTTATATACTGTTTTTGGCATGACTTATATATATACACAAAAAATCACCTAACTGCACATTCCAAATCCAAACACCACACCACAAAAAAAATAACAATAACAATA  5699
                   ......5560......5570......5580......5590......5600......5610......5620......5630......5640......5650......5660......5670......5680......5690......5700

                   ******************************************************************************************************************************************************
TEfam_Outcast_Ele6 ATAAAATTTAATAAATTCAATAATAAATCCGATTTTACACGCATCAGGGGGGCCTCCTAAGTTTGGGTACTCCGTTTGTTCTTGCAAACATGAGAAATCCAAAGACATCCATTTCCATCACTAGAGGATAGAAATAAGATGCCAAAAGGA  5830
TESeeker_Outcast   ATAAAATTTAATAAATTCAATAATAAATCCGATTTTACACGCATCAGGGGGGCCTCCTAAGTTTGGGTACTCCGTTTGTTCTTGCAAACATGAGAAATCCAAAGACATCCATTTCCATCACTAGAGGATAGAAATAAGATGCCAAAAGGA  5849
                   ......5710......5720......5730......5740......5750......5760......5770......5780......5790......5800......5810......5820......5830......5840......5850

                   ***************************************************   ******************* **:*************************************************************************
TEfam_Outcast_Ele6 CCAACTGAAAGGGCCTTGTATCAAATCCCCCCCCCCCAAAAAAAAAAAAAA---TCCAAAATAAAAAAAAAAA-AATAAAAAAAAAAACCCTTTTGGAACCTTCACAAAACAAGCCATTTCAAAAATAATAATAATAAAAATAAAAATAA  5976
TESeeker_Outcast   CCAACTGAAAGGGCCTTGTATCAAATCCCCCCCCCCCAAAAAAAAAAAAAAAAATCCAAAATAAAAAAAAAAATAAAAAAAAAAAAAACCCTTTTGGAACCTTCACAAAACAAGCCATTTCAAAAATAATAATAATAAAAATAAAAATAA  5999
                   ......5860......5870......5880......5890......5900......5910......5920......5930......5940......5950......5960......5970......5980......5990......6000

                   ********************************************   ********************************:***********************   ******************************************* 
TEfam_Outcast_Ele6 ACAACCCAATACAAGAACAAGAACCGGTACAAAAAAAAAAAAAAAAACAAAATTCCTCATCCTCTCCCAAGAAATAAAAAATATATATATATATACAAACACCACAAAAAAAAAAAAAGAAGAAAATCAAATCCACTAAACCACAAGCGAAT  6126
TESeeker_Outcast   ACAACCCAATACAAGAACAAGAACCGGTACAAAAAAAAAAAAAA---CAAAATTCCTCATCCTCTCCCAAGAAATAAAATATATATATATATACAAACACCAC---AAAAAAAAAAGAAGAAAATCAAATCCACTAAACCACAAGCGAAC  6143
                   ......6010......6020......6030......6040......6050......6060......6070......6080......6090......6100......6110......6120......6130......6140......6150

                   ******************************************************************************************************************************************************
TEfam_Outcast_Ele6 TTAAAGATGGAACAACGCTAGCTTTTGAATACAAACTGAAAAGGAAACACAAACCATCAAAAAGAAAAAACGATTGGTGATATCACGAAAACCAACACACTGTGCATCAATCAGAAAGATCTCAACCACAACATATAAACATCAACGAAA  6276
TESeeker_Outcast   TTAAAGATGGAACAACGCTAGCTTTTGAATACAAACTGAAAAGGAAACACAAACCATCAAAAAGAAAAAACGATTGGTGATATCACGAAAACCAACACACTGTGCATCAATCAGAAAGATCTCAACCACAACATATAAACATCAACGAAA  6293
                   ......6160......6170......6180......6190......6200......6210......6220......6230......6240......6250......6260......6270......6280......6290......6300

                   ************************************************************************************************************************************
TEfam_Outcast_Ele6 CAGCAATATAAACATCAAAGGAGAATGGATGATTTCCCCTCCAAAAACTGGTAGTCAAACCTCTGATTGTACCTTCAAAAACGGATGGTATAGGAGGCCTCAACCTACCAACCCTATTAAGAAGAAGAAGAA-----------  6408
TESeeker_Outcast   CAGCAATATAAACATCAAAGGAGAATGGATGATTTCCCCTCCAAAAACTGGTAGTCAAACCTCTGATTGTACCTTCAAAAACGGATGGTATAGGAGGCCTCAACCTACCAACCCTATTAAGAAGAAGAAGAAGAAGAAAACGG  6436
                   ......6310......6320......6330......6340......6350......6360......6370......6380......6390......6400......6410......6420......6430......6440...
```

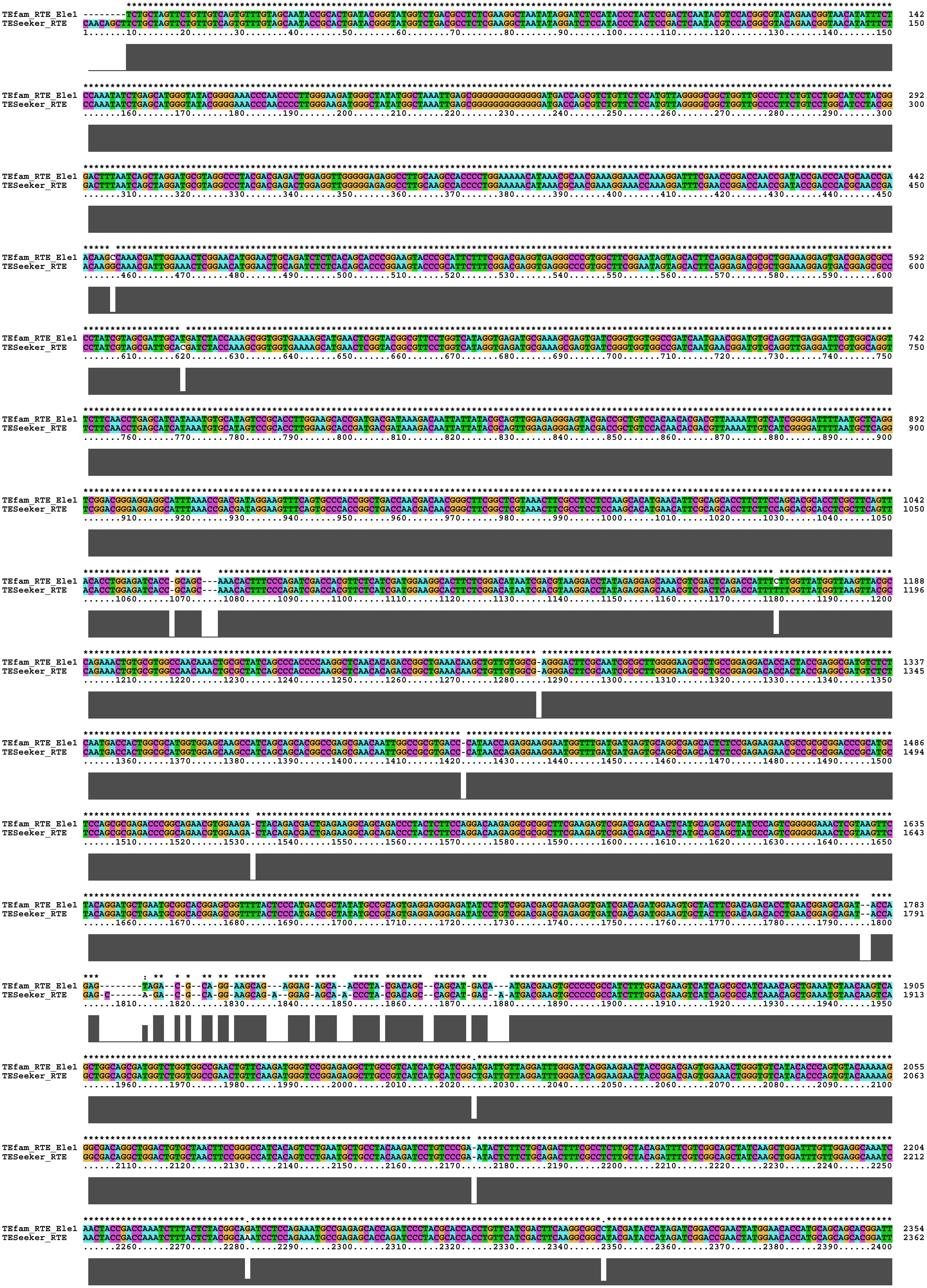

TEfam_RTE_Ele1
TESeeker_RTE

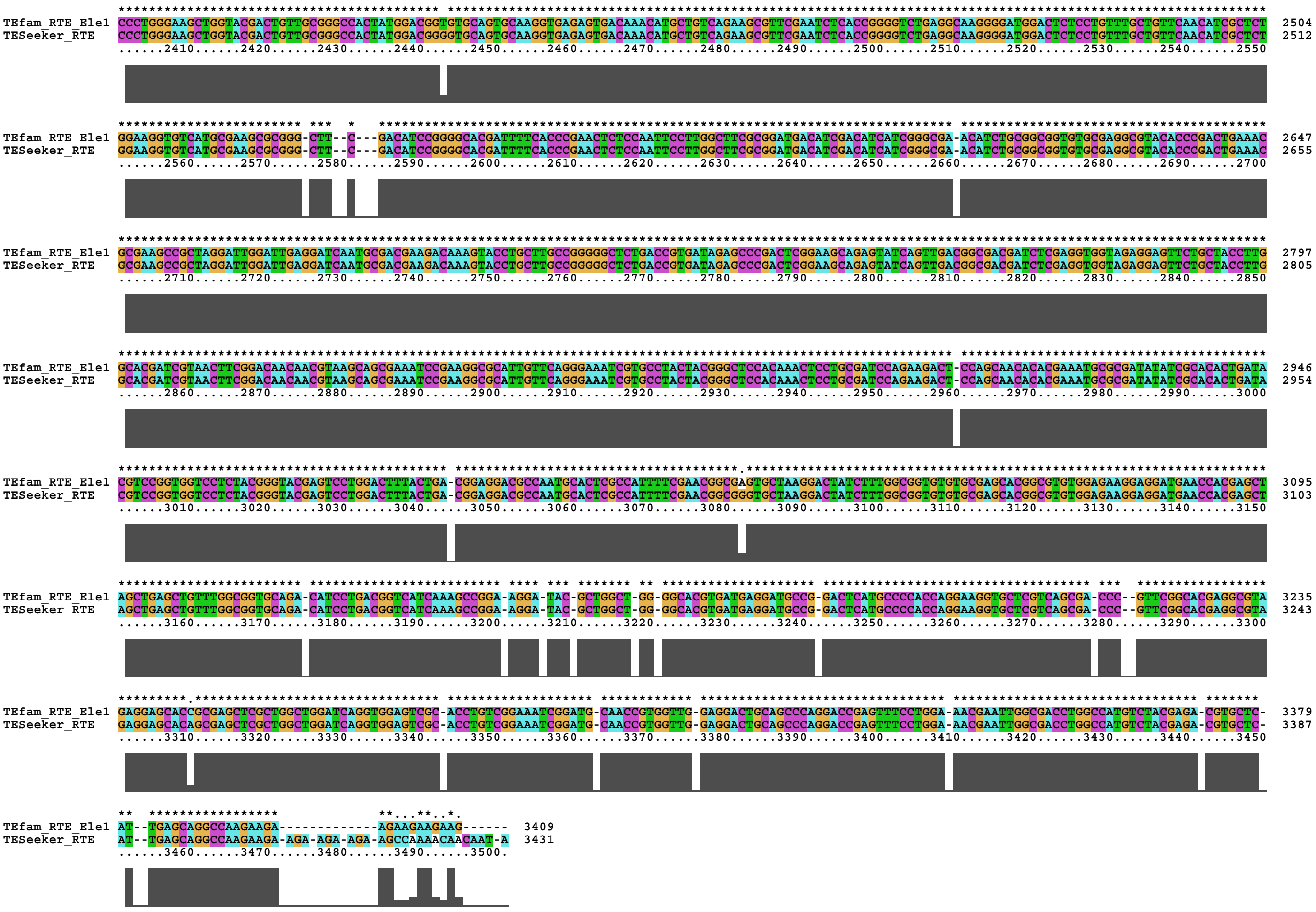
TEfam_RTE_Ele1
TESeeker_RTE
