# Supplementary material for: An automated homology-based approach for identifying transposable elements
Source: BMC Bioinformatics. 2011 May 3;12:130. doi: 10.1186/1471-2105-12-130 (PMC3107183; doi:10.1186/1471-2105-12-130)
Supplement: Additional file 3 — Annotated putative D. melanogaster mariner element. [file 1471-2105-12-130-S3.PDF]

Additional File 3

Annotated putative *mariner* element from *Drosophila melanogaster*.

```
DEFINITION           Putative Drosophila melanogaster mariner sequence
SOURCE               Drosophila melanogaster (fruit fly)
                     flybase.org, dmel-all-chromosome-r5.29.fasta
FEATURES             Location/Qualifiers
     source          1..1043
                     /organism="Drosophila melanogaster"
                     /mol_type="genomic DNA"
                     /transposon="putative mariner transposon"
     repeat_region   1..26
                     /note="left terminal inverted repeat"
     ORF1            66..780
                     translation=LCNCILSSVSYQLLLLAECQNWFRKFRSGDFSLKHEPRSGRLYEV
                     DDDLIKALIELDRHVNKQEIGEKFNIPKSTVYYHIKRLVKKFDIWVPHVLKEIHLTH
                     RINACDMQLKCNEFDPFLKRITSGKEKWIVYNNVSRKRSWSKHGEPAQTTSKADIHQ
                     KKVMLSVWWDWKGVVYFELLPRNQTINSDVYCHQLNKLNTRRSDQNWSIVKVSYSTR
                     ITLDCTHLWSLSKNCVSLGRNF
                     /product="putative mariner transposase"
     repeat_region   1018..1043
                     /note="right terminal inverted repeat"
ORIGIN
        1  TGCCCAAAAA GTAATTGCGG ATTTTTCATA TAGTCGGCGT TGACAAATTT
       51  TTTCAACGGC TTGTGACTTT GTAATTGCAT TCTTTCATCT GTCAGTTATC
      101  AGCTGTTACT ATTAGCTGAG TGTCAAAATT GGTTTCGCAA ATTCCGTTCT
      151  GGAGATTTTT CACTTAAACA TGAGCCCCGT TCAGGTCGGC TATATGAAGT
      201  TGATGATGAC CTAATCAAAG CATTAATCGA ATTGGATCGT CATGTAAATA
      251  AGCAGGAGAT AGGAGAGAAG TTTAATATAC CAAAATCAAC CGTTTACTAT
      301  CACATAAAAA GACTAGTGAA AAAGTTTGAT ATTTGGGTAC CACATGTATT
      351  GAAAGAAATT CATTTAACAC ACCGAATAAA TGCTTGTGAT ATGCAACTTA
      401  AATGCAATGA ATTCGATCCG TTTTTAAAAC GAATCACATC TGGAAAGGAA
      451  AAATGGATTG TTTACAACAA CGTTAGTCGA AAACGATCAT GGTCCAAGCA
      501  TGGTGAACCA GCTCAAACCA CTTCAAAGGC TGATATCCAC CAAAAGAAGG
      551  TTATGCTGTC TGTTTGGTGG GATTGGAAGG GTGTCGTATA TTTTGAACTG
      601  CTTCCAAGGA ACCAAACGAT TAATTCGGAT GTTTACTGTC ACCAATTGAA
      651  CAAATTGAAT ACAAGGAGAA GCGACCAGAA TTGGTCAATC GTAAAGGTGT
      701  CATATTCCAC CAGGATAACG CTAGACTGCA CACATCTTTG GTCACTATCC
      751  AAAAACTGTG TGAGCTTAGG TAGGAACTTT TGATGCATCC ACCGTATAGC
      801  CCTGACCTGG AACCATCAGA CTACCATTTA TTTCGATCTT TGCAGAACTC
      851  CTTAAATGGT AAAACTTTCG GGAATGATGA GGCTATAAAA TCGCACTTGG
      901  TTCAGTTTTT TGCAGATAAA GGCCAGAAGT TCTATTGACC GTGGAATAGA
      951  AAAAAGGTTA TCGAAAAAAA TGGCAATTCA TTCTAAGTAT TATTAAAAAT
     1001  GCATTTACTT TCTTTTAAAA AATCGGAAAT TATTTTTTGG GCA
//
```
