# Supplementary material for: An automated homology-based approach for identifying transposable elements
Source: BMC Bioinformatics. 2011 May 3;12:130. doi: 10.1186/1471-2105-12-130 (PMC3107183; doi:10.1186/1471-2105-12-130)
Supplement: Additional file 4 — TESeeker User Manual. [file 1471-2105-12-130-S4.PDF]

Additional File 4

`TESeeker` User Manual.

TESeeker User Manual v1.04

Ryan C. Kennedy
Department of Computer Science and Engineering
University of Notre Dame
Ryan.C.Kennedy@alumni.nd.edu

April 20, 2011

1 Background

Transposable elements (TEs) are a type of repetitive sequence that have been found in nearly all eukaryotic genomes. First discovered and analyzed by McClintock in the 1950s [7], TEs have the ability to move about and replicate within a genome. Due to their mobile and replicative nature, TEs often occupy large portions of genomes. This prevalence of TEs poses a major difficulty in sequence assembly, as repeat regions are prone to misassembly [9, 10]. TEs can impact host genomes in a number of ways. They are believed to play a major role in genome evolution [4, 8, 12], as they can insert themselves into, mutate, and move genes, thereby influencing gene expression, causing gene variation, and transferring genetic material [1, 2, 11, 13].

The number of sequenced genomes is rapidly rising, and the need to identify TEs within them is also growing. The ability to do this automatically and effectively in a manner similar to the methods used for genes is of increasing importance. This document describes how to use the implementation of our approach, `TESeeker`, to identify high-quality consensus TEs.

2 Requirements

`TESeeker` is available as a `VirtualBox` [16] virtual appliance in the open virtualization format (OVF). `TESeeker` requires at least 5 GB free hard disk space and at least 1.5 GB of RAM on the host machine. `TESeeker` can dynamically allocate up to 40 GB hard disk space for use in the virtual appliance. `TESeeker` is licensed under GNU General Public License (GPL) v3 [3].

3 Installation

`TESeeker` can run on any operating system that supports the VirtualBox virtualization software package, currently available for Windows, OS X, Linux, and

Solaris.

The following steps shall be followed to install TESeeker:

1. Download and install VirtualBox from:
   http://www.virtualbox.org.
2. Download the TESeeker virtual appliance files (2) from:
   http://repository.library.nd.edu/view/27/teseeker.
3. Open VirtualBox.
4. Click *File* then *Import Appliance...* and complete the wizard, selecting the TESeeker .ovf file as the source. Be sure both downloaded TESeeker files are in the same directory.

4 Usage

After installation, start TESeeker by opening VirtualBox, clicking *teseeker* in the left frame, and then clicking *Start*. The virtual appliance hosting TESeeker will then boot.[1] As shown in Figures 1-7, the booted appliance will contain 7 desktop items: the *Genomes* and *TELibrary* folders, shortcuts to bring up the documentation and web interfaces, and the license. The TESeeker interface is shown in Figure 5. Hovering the mouse over the parameter name will provide a more detailed description. All genomes and library files must be placed in the folders on the desktop and must be in the FASTA file format with a .fa, .fas, or .fasta file extension. We have included the *Pediculus humanus humanus* genome and our representative TE library within the virtual appliance.

Clicking the *TESeeker* shortcut on the desktop will load the web interface. Here, researchers can modify the default parameters, most notably the *BLAST Query Library*, *BLAST Database*, and the *Desktop Output Folder Name.* Hovering over the parameter name will provide a detailed tooltip description. Once the parameters have been set, clicking *submit* will briefly show the selected parameters and then start the search. The browser will display *Job X is Running*, where X represents the job identification number. The browser will continually refresh the page until the job completes, at which point the page will notify the user. When finished, researchers navigate to the specified output folder on the desktop to view results.

If the researcher elects to find only the coding region, results are organized as follows within the specified output folder: the *codingRegion_files* folder contains intermediary output, the *output* folder contains all the singlet and contig sequences produced, and the remaining files represent the singlet and contig sequences produced from CAP3. For example, a file called *cap2c_out.fas* contains the contig sequences from the second iteration of CAP3, while *cap1s_out.fas* contains the singlet sequences produced from the first iteration of CAP3.

[1] Some Linux distributions automatically enable the *KVM kernel extension* If this is the case, disable it with the following command sudo modprobe -r kvm_intel. To restore the *KVM kernel extension*, run sudo modprobe kvm_intel.

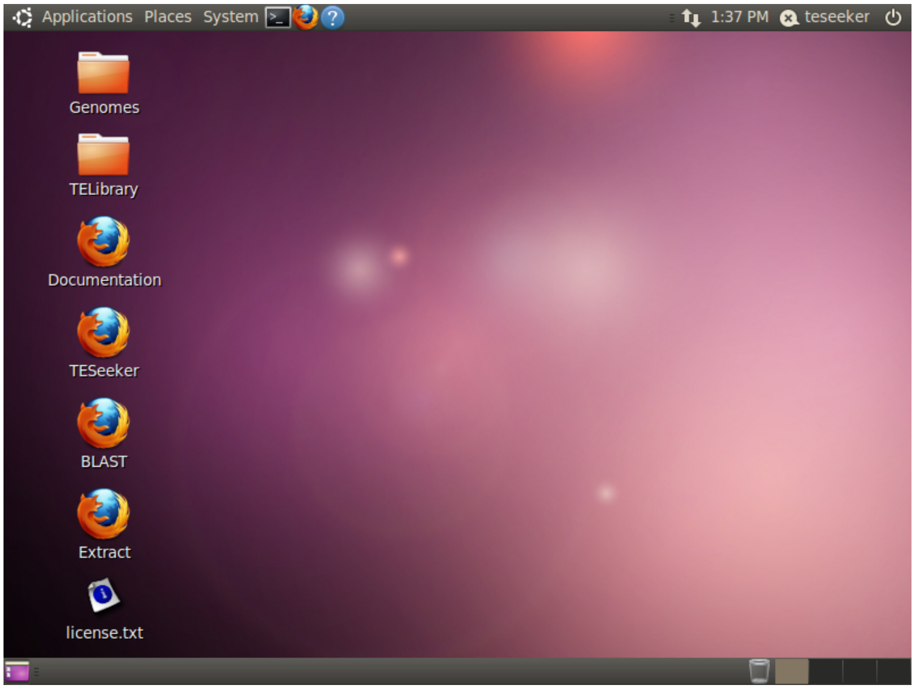

Figure 1: `TESeeker` Desktop. This figure shows the desktop.

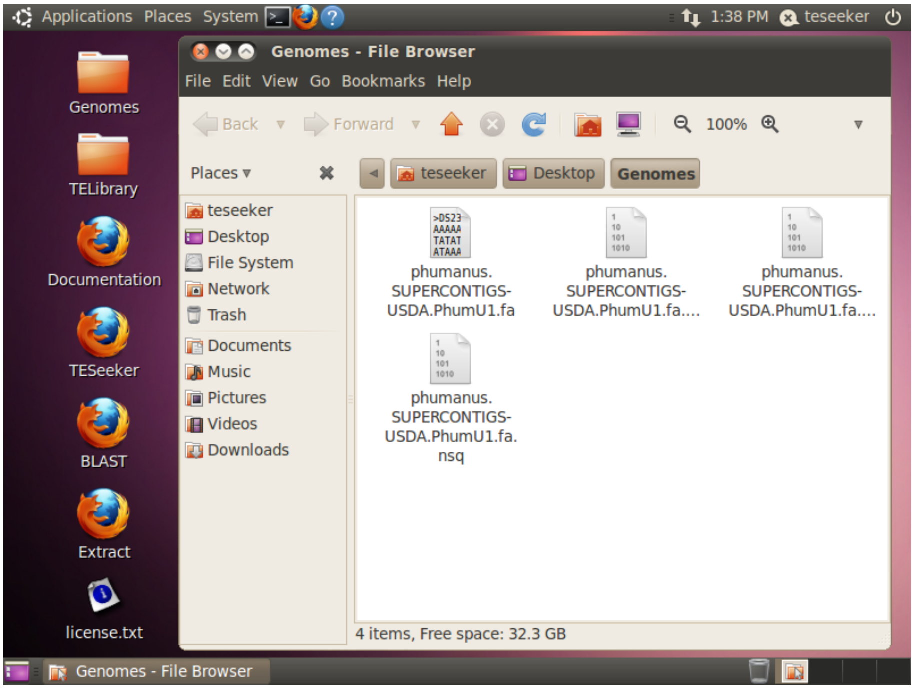

Figure 2: `TESeeker` *Genomes* Folder. Researchers can place FASTA genome data in this folder.

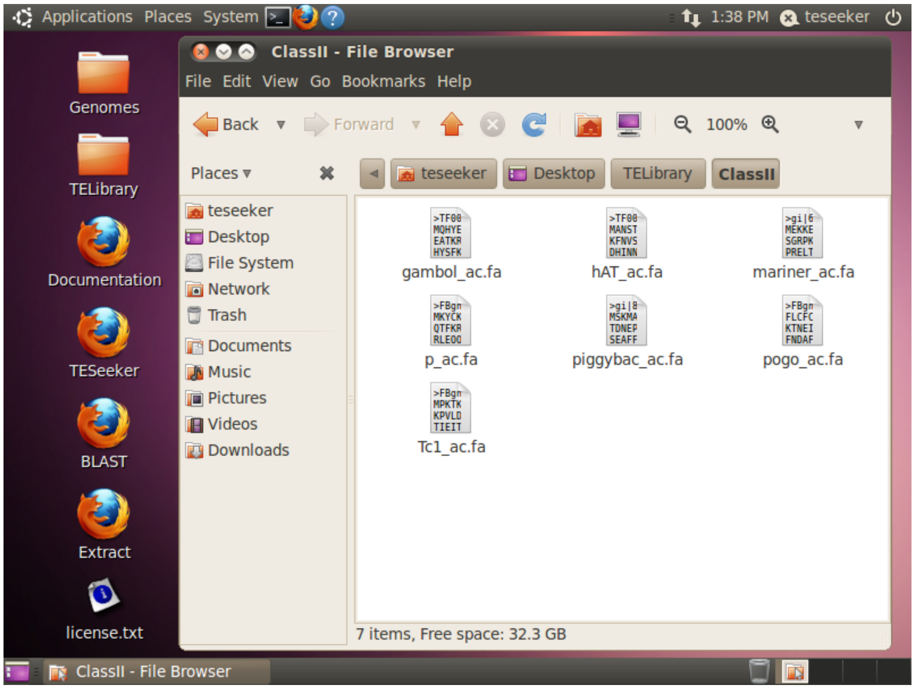

Figure 3: `TESeeker` *TELibrary*. This figure shows the folder for the Class II representative TEs. Researchers can also place additional FASTA sequence data in the class-appropriate folders.

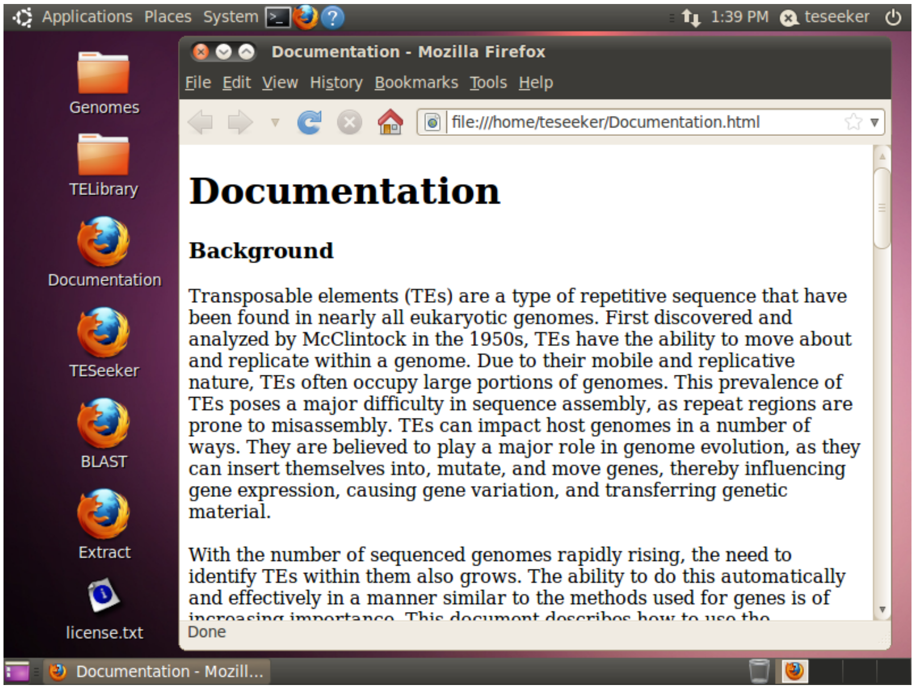

Figure 4: `TESeeker` Documentation. Here, we show a screen capture of the HTML `TESeeker` Documentation.

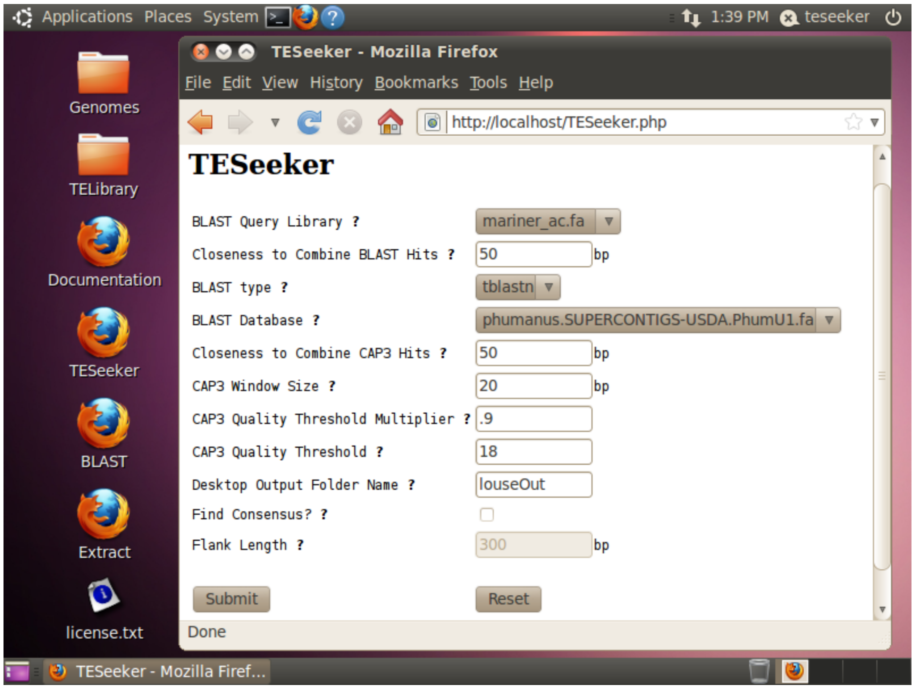

Figure 5: `TESeeker` Web Interface. This figure shows a portion of the `TESeeker` web interface. Researchers can alter the default parameters as desired. Library and genome files in the desktop folders are selectable through drop-down menus.

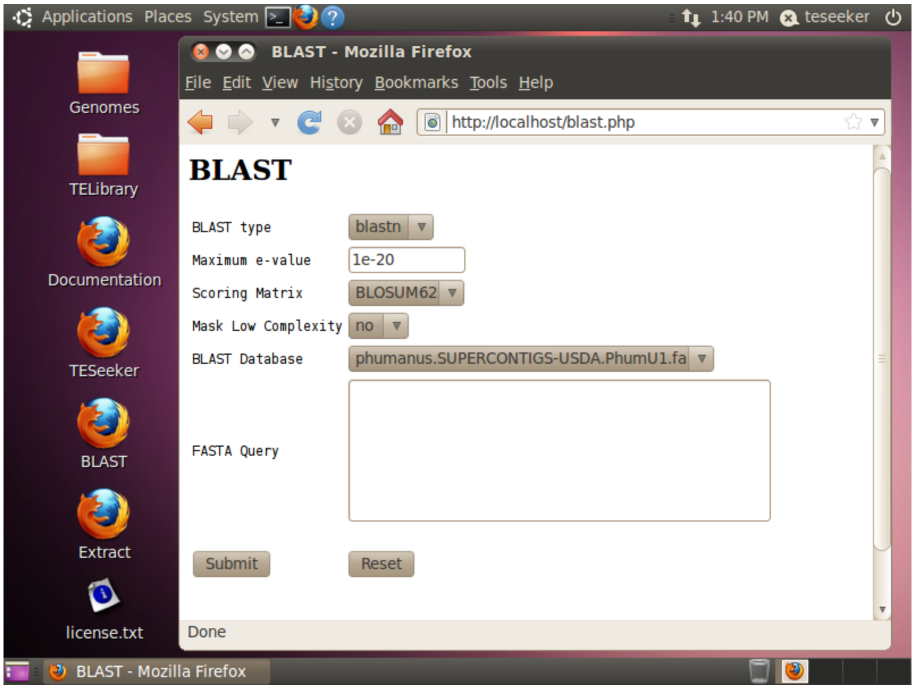

Figure 6: `TESeeker` BLAST Interface. Here, we show the BLAST interface. The BLAST Database drop-down menu is populated via the genomes available in the *Genomes* folder.

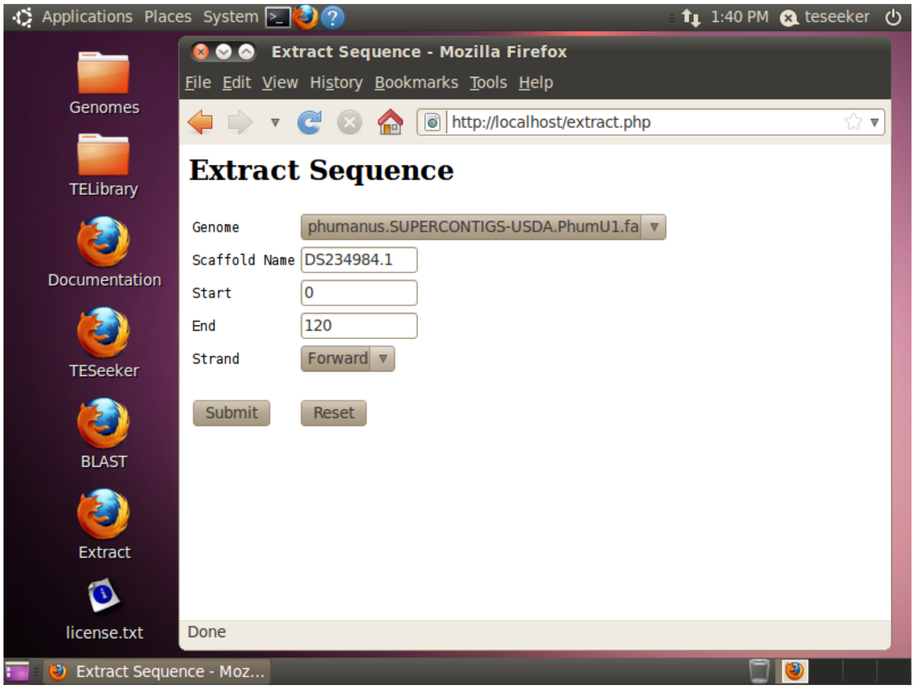

Figure 7: `TESeeker` Extract Interface. This figure shows the `TESeeker` extract interface. Researchers can extract specified sequence data from any genome in the *Genomes* folder.

If a consensus sequence is desired, the results are organized as follows within the specified output folder: the *codingRegion_files* folder contains intermediary output from the coding region search, the folder *consen_files* contains intermediary files from the consensus search, and the *output* folder contains the contig and singlet sequences produced from each sequence that was fed into the consensus search. Additionally, all contig and singlet sequences are available in single FASTA files in the specified output folder.

5 Example Search

`TESeeker` is distributed with the *Pediculus humanus humanus* genome as well as our library of representative TEs. We next describe how one could obtain a high-quality consensus element for the *Pediculus humanus humanus mariner* element, once the virtual appliance has been loaded.

1. **Launch `TESeeker`**. Double-click the `TESeeker` shortcut on the desktop.

2. **Confirm Parameters**. Ensure *mariner_ac.fa* is selected for the *BLAST Query Library* and that the *phumanus.SUPERCONTIGS-USDA.PhumUA.fa* genome is selected for the *BLAST database*. Also click *Find Consensus?* to enable a consensus search. The screen should now look as shown in Figure 8. The status for `TESeeker` will be continuously updated through the web interface until the job completes.

3. **Inspect Results**. When the job is finished, click the link to the specified output folder, *louseOut*, and inspect the results. The web view of this folder is shown in Figure 9. As mentioned in the previous section, the main consensus results will be in up to three FASTA files, *consensus_contigs.fas*, *consensus_iter1_singlets.fas*, and *consensus_singlets.fas.* The best hits are generally in the *consensus_contigs.fas* file, while the ones with the least likelihood are generally in the *consensus_iter1_singlets.fas* file. In this case, the first contig in *consensus_contigs.fas*, *Contig1-0 6 1309 f*, contains a sequence 99% identical to the manually annotated element, differing mainly in its roughly 10 extra nucleotides on both ends. Figure 10 shows the ends of the aligned sequences.

6 Additional Tools

There are also *BLAST* and *Extract* shortcuts on the desktop. These web interfaces offer additional functionality by making it straightforward to do a custom BLAST search or sequence extraction using the files in the *Genomes* folder.

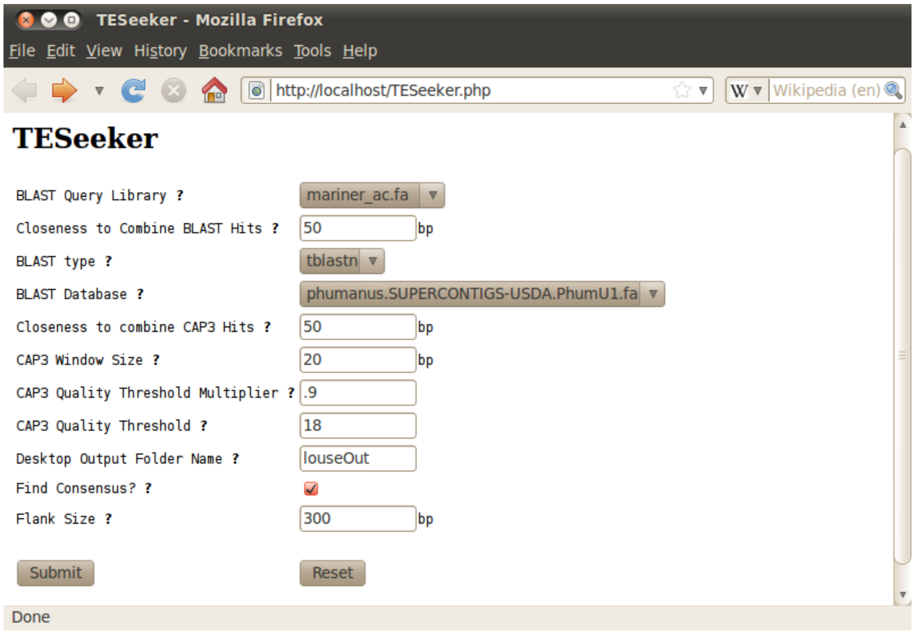

Figure 8: `TESeeker` Default Parameters. This figure shows the `TESeeker` web interface with the default parameters set for a search for the *mariner* transposon in *P. humanus humanus*.

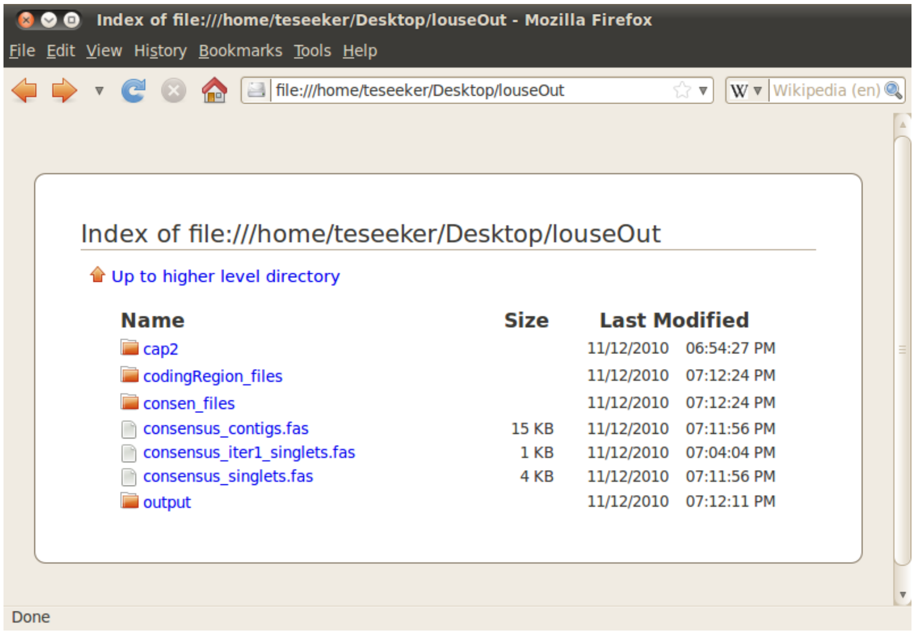

Figure 9: Web Interface File Browser. The figure above shows the contents of the main output folder, *louseOut*. FASTA sequences are in the `.fas` files, shown here as *consensus_contigs.fas*, *consensus_iter1_singlets.fas*, and *consensus_singlets.fas*.

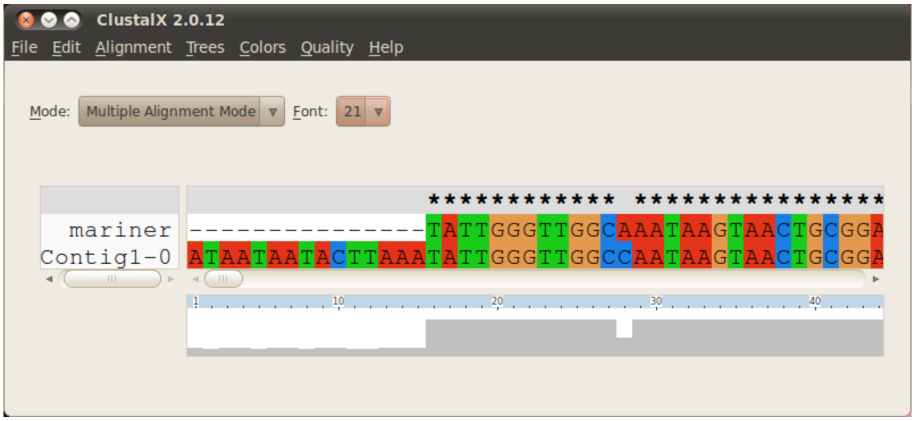

(a) 5' End

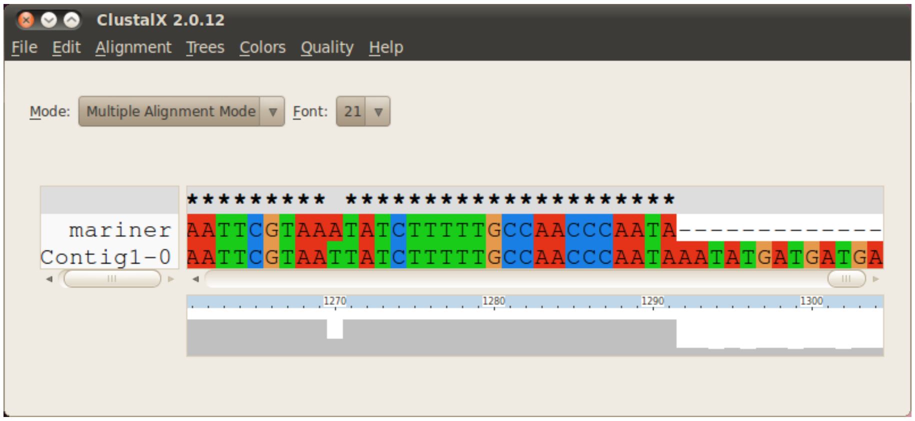

(b) 3' End

Figure 10: `ClustalX` Alignment with Annotated Element. Panels (a) and (b) show the 5' and 3' ends of the annotated *mariner* (mariner) and the top consensus sequence produced by `TESeeker` (Contig1-0) when run with the default parameters. The sequences are 99% identical. The extra sequence on both ends of Contig1-0 can be reduced with stricter parameters.

7 Technology

`TESeeker` utilizes a variety of technologies. The core bioinformatics tools, BLAST, `CAP3`, `ClustalW2`, and BioPerl, are united through bash scripts. Researchers interact with `TESeeker` through a web-based form implemented in html/php and handled by the `lighttpd` web server. The form interacts with the local scripts and utilizes a PostgreSQL database and cgi/Perl to notify researchers when a job has completed. `TESeeker` is installed on Ubuntu 10.04 LTS. The administrative password for user teseeker is `teseeker`.

8 Acknowledgments

Development of `TESeeker` was supported in part by NIAID/NIH contracts HHSN272200900039C and HHSN266200400039C for "VectorBase: A Bioinformatics Resource Center for Invertebrate Vectors of Human Pathogens" [5,6,15]. LTR reverse transcriptases within the representative library were chosen with the assistance of Jose Manuel C. Tubío. Computational resources provided in part by the Notre Dame Center for Research Computing [14].

References

[1] E. A. Bennett, L. E. Coleman, C. Tsui, W. S. Pittard, and S. E. Devine. Natural genetic variation caused by transposable elements in humans. *Genetics*, 168:933–951, October 2004.

[2] N. L. Craig, R. Craigie, M. Gellert, and A. M. Lambowitz, editors. *Mobile DNA II.* ASM Press, Washington, DC, 2002.

[3] GNU General Public License (GPL) v3. http://www.gnu.org/licenses/gpl.html.

[4] M. G. Kidwell and D. Lisch. Transposable elements as sources of variation in animals and plants. *Proceedings of the National Academy of Sciences USA*, 94:7704–7711, July 1997.

[5] D. Lawson, P. Arensburger, P. Atkinson, N. J. Besansky, R. V. Bruggner, R. Butler, K. S. Campbell, G. K. Christophides, S. Christley, E. Dialynas, D. Emmert, M. Hammond, C. A. Hill, R. C. Kennedy, N. F. Lobo, R. M. MacCallum, G. Madey, K. Megy, S. Redmond, S. Russo, D. W. Severson, E. O. Stinson, P. Topalis, E. M. Zdobnov, E. Birney, W. M. Gelbart, F. C. Kafatos, C. Louis, and F. H. Collins. VectorBase: a home for invertebrate vectors of human pathogens. *Nucleic Acids Research*, 35:D503–D505, 2007.

[6] D. Lawson, P. Arensburger, P. Atkinson, N. J. Besansky, R. V. Bruggner, R. Butler, K. S. Campbell, G. K. Christophides, S. Christley, E. Dialynas, M. Hammond, C. A. Hill, N. Konopinski, N. F. Lobo, R. M. MacCallum,

G. Madey, K. Megy, J. Meyer, S. Redmond, D. W. Severson, E. O. Stinson, P. Topalis, E. Birney, W. M. Gelbart, F. C. Kafatos, C. Louis, and F. H. Collins. VectorBase: a data resource for invertebrate vector genomics. *Nucleic Acids Research*, 37:D583–587, 2009.

[7] B. McClintock. *The discovery and characterization of transposable elements: The collected papers of Barbara McClintock.* Garland Publishing, Inc., New York, NY, 1987.

[8] P. Medstrand, L. N. van de Lagemaat, C. A. Dunn, J. R. Landry, D. Svenback, and D. L. Mager. Impact of transposable elements on the evolution of mammalian gene regulation. *Cytogenetic and Genome Research*, 110:342–352, 2005.

[9] J. R. Miller, S. Koren, and G. Sutton. Assembly algorithms for next-generation sequencing data. *Genomics*, 95(6):315 – 327, 2010.

[10] M. Pop, S. L. Salzberg, and M. Shumway. Genome sequence assembly:algorithms and issues. *Computer*, 35:47–54, 2002.

[11] J. A. Shapiro. The discovery and significance of mobile genetic elements. In D. J. Sherratt, editor, *Mobile Genetic Elements.* Oxford University Press, 1995.

[12] R. K. Slotkin and R. Martienssen. Transposable elements and the epigenetic regulation of the genome. *Nature Reviews Genetics*, 8(4):272–285, April 2007.

[13] Z. Tu and C. Coates. Mosquito transposable elements. *Insect Biochemistry and Molecular Biology*, 34:631–644, 2004.

[14] University of Notre Dame Center for Research Computing. http://crc.nd.edu.

[15] VectorBase: A Bioinformatics Resource Center for Invertebrate Vectors of Human Pathogens. http://www.vectorbase.org.

[16] VirtualBox. http://www.virtualbox.org.
